# Episodic Memory and Recollection Network Disruptions Following Chemotherapy Treatment in Breast Cancer Survivors: A Review of Neuroimaging Findings

**DOI:** 10.3390/cancers14194752

**Published:** 2022-09-29

**Authors:** Meenakshie Bradley-Garcia, Gordon Winocur, Melanie J. Sekeres

**Affiliations:** 1 School of Psychology, University of Ottawa, Ottawa, ON K1N 6N5, Canada; 2 Rotman Research Institute, Baycrest Centre, Toronto, ON M6A 2E1, Canada; 3 Department of Psychology, Department of Psychiatry, University of Toronto, Toronto, ON M5S 3G3, Canada; 4 Department of Psychology, Trent University, Peterborough, ON K9J 7B8, Canada

**Keywords:** cognitive impairment, memory loss, breast cancer, chemotherapy, neuroimaging, medial temporal lobe, hippocampus

## Abstract

**Simple Summary:**

Memory disturbances are amongst the most common and disruptive symptoms of chemotherapy-related cognitive impairment. Chemotherapy treatments commonly cause neurotoxicity within the hippocampus, creating a vulnerability to memory impairment. Most clinical assessments of long-term memory in breast cancer survivors assess basic verbal and visual memory processing, and do not capture the complexities of everyday event memories, including episodic and autobiographical memory. This review focuses on structural and functional neuroimaging studies identifying disruptions in the hippocampus and recollection network, and related episodic memory impairments in chemotherapy-treated breast cancer survivors. We argue for the need to better characterize memory dysfunction following chemotherapy treatments. Given the importance of episodic and autobiographical memory to a person’s personal history and quality of life, an under-appreciation of how this memory domain is impacted by standard cancer treatments potentially diminishes the negative experiences of breast cancer survivors, and neglects cognitive problems that could benefit from intervention strategies.

**Abstract:**

Long-term memory disturbances are amongst the most common and disruptive cognitive symptoms experienced by breast cancer survivors following chemotherapy. To date, most clinical assessments of long-term memory dysfunction in breast cancer survivors have utilized basic verbal and visual memory tasks that do not capture the complexities of everyday event memories. Complex event memories, including episodic memory and autobiographical memory, critically rely on hippocampal processing for encoding and retrieval. Systemic chemotherapy treatments used in breast cancer commonly cause neurotoxicity within the hippocampus, thereby creating a vulnerability to memory impairment. We review structural and functional neuroimaging studies that have identified disruptions in the recollection network and related episodic memory impairments in chemotherapy-treated breast cancer survivors, and argue for the need to better characterize hippocampally mediated memory dysfunction following chemotherapy treatments. Given the importance of autobiographical memory for a person’s sense of identity, ability to plan for the future, and general functioning, under-appreciation of how this type of memory is impacted by cancer treatment can lead to overlooking or minimizing the negative experiences of breast cancer survivors, and neglecting a cognitive domain that may benefit from intervention strategies.

## 1. Introduction

Advances in diagnostic and therapeutic interventions for breast cancer have led to high patient survival rates [1]. The return to normal daily activities following cancer treatment is often hampered by treatment-related side effects that impact cognitive function [1,2]. In the months and years following treatment, up to 75% of women successfully treated with chemotherapeutic agents for breast cancer experience chemotherapy-related cognitive impairment (CRCI), or ‘chemobrain’, described by patients as a feeling of fuzzy headedness or mental slowness [3,4,5]. The most commonly observed symptoms, as assessed through neurocognitive testing, are long-term memory loss, attentional difficulties, and impaired executive functioning that affects planning, problem solving, and working memory [1,4,6,7,8,9]. These cognitive changes severely disrupt survivors’ ability to carry out normal daily activities [4,7,10,11,12], and have a significant impact on overall quality of life. CRCI has been observed up to 20 years following treatment [3,13,14,15], with structural and functional differences evident in the brain for at least 10 years post-treatment [16,17,18,19]. These findings highlight the long-lasting and pervasive impact on the neural physiology and well-being of survivors [3].

CRCI has been seen in many other types of non-CNS cancers [20] but is most prevalent and most studied in breast cancer. Long-term memory disturbances are amongst the most common and disruptive symptoms experienced by breast cancer survivors following chemotherapy, yet the physiological mechanisms underlying disrupted memory processing following chemotherapy are not well characterized [21]. This narrative review and commentary focuses specifically on chemotherapy-related memory impairments in breast cancer survivors, including the largely neglected domain of autobiographical memory. We discuss potential neural mechanisms contributing to memory processing deficits, with a focus on structural (MRI) and functional (fMRI) neuroimaging studies identifying alterations in the hippocampus and related medial-temporal lobe structures in breast cancer survivors.

We searched the PubMed and Google Scholar data bases between 2000 and 2022 using the search terms ‘chemotherapy’, ‘breast cancer’, ‘chemofog’, ‘chemotherapy-induced cognitive impairment’, ‘episodic memory’ ‘long-term memory’, ‘autobiographical memory’, ‘resting state’, ‘default mode network’, ‘hippocampus’, ‘temporal lobe’, ‘MRI,’ ‘fMRI’. Papers were excluded if they did not include measures of long-term memory (delayed verbal memory, delayed visual memory, episodic memory, autobiographical memory) or structural or functional assessments of the temporal lobes or recollection/default mode network in chemotherapy-treated breast cancer survivors.

## 2. Physiological Mechanisms Contributing to Chemotherapy-Related Memory Disruption

The hippocampus, a medial-temporal lobe structure that is critical for memory processing, has been found to be particularly sensitive to structural and functional disruption following chemotherapy treatment [22]. The physiological mechanisms mediating these disruptions and related cognitive impairments are multifactorial, including breakdown of the blood–brain barrier, pro-inflammatory cytokine release (IL-6, IL-1B, TNF-α) [23], increases in reactive oxidative stress and mitochondrial dysfunction [24,25], enhanced activated microglia [26,27], neuronal morphology abnormalities including reduced dendritic branching and spine density in the hippocampus [27,28,29], and white matter microstructural changes, and reduced gray matter volume throughout the brain [30,31]. While these factors likely combine to exacerbate the broader cognitive dysfunction characterizing CRCI, a likely candidate mediating long-term memory loss following chemotherapy is a reduction in adult hippocampal **neurogenesis**. 

Hippocampal subregions (CA1, CA3, and dentate gyrus) have specialized functions, with the dentate gyrus being of particular interest in understanding chemotherapy-related cognitive impairment and neurotoxicity due to its role in neurogenesis. The dentate gyrus is unique, in that it is one of two known regions to continually generate new neurons in the mammalian brain [32]. This process of hippocampal neurogenesis contributes to a renewing pool of neurons that functionally incorporate into new memory networks [33], and critically contribute to the process of memory consolidation [34], memory clearance [35,36], and cognitive flexibility [37]. Experimentally induced suppression of adult neurogenesis typically results in deficits on hippocampally mediated memory tasks in animal models [38,39,40,41]. 

Several molecular mechanisms referred to above have been linked to reduced rates of hippocampal neurogenesis. For example, cell damage induced by chemotherapy-induced increases in reactive oxidative stress reduces the survival of primary neural precursor cells and inhibits the production of new cells in the hippocampus [24,25,42,43]. Similarly, stress-induced expression of pro-inflammatory cytokines IL-6 and TNF-α suppresses doublecortin levels within the hippocampus, a cellular marker expressed by immature neurons [44].

The range of systemic chemotherapeutic agents commonly used in breast cancer therapy have been shown to suppress hippocampal neurogenesis and to impair hippocampally mediated memory in rodents, including the anti-metabolites methotrexate [45,46,47,48], 5-FU [49,50,51,52], cisplatin [53,54,55], alkylating agents cyclophosphamide [8,26,56,57], temozolomide [58,59,60,61], mitotic inhibitors doxorubicin [8,26,62] and paxlitaxel [50,63,64,65], both when used alone or in combination [29,39,66,67,68,69,70,71,72] (see Sekeres et al. [72] for extensive review of the classes of chemotherapy drugs and their effects on hippocampal neurogenesis and memory performance in pre-clinical models). These findings provide strong evidence that the neurotoxic effects of common breast cancer treatments are sufficient to induce cell-specific hippocampal neurotoxicity that, in part, mediates long-term memory deficits observed in patients. 

In vivo structural neuroimaging studies in humans lack the spatial resolution to assess differences in dentate gyrus volume at the cellular level, and cannot distinguish natally generated neurons from adult-generated neurons. However, hippocampal segmentation analyses have identified differences in hippocampal sub-region volume between chemotherapy-treated breast cancer survivors and healthy controls, suggesting that systemic chemotherapy treatments are capable of inducing changes in the human hippocampal architecture [73]. Post-mortem observations in human brain tissue have confirmed that common cancer treatments (systemic chemotherapy, cranial radiation,) are capable of suppressing hippocampal neurogenesis [74].

Given the role of the hippocampus in memory processing, understanding changes in hippocampal integrity following various chemotherapy treatments is essential to understanding the associated memory impairments in breast cancer survivors. Quantifying adult hippocampal neurogenesis remains a challenge in humans [75,76]. There is post-mortem evidence that adult hippocampal neurogenesis persists throughout the lifespan in humans, though some age-related declines in neurogenic rates are evident, particularly within the anterior hippocampus [77,78,79]. Treatments that impair the normal proliferation and survival rates of adult generated hippocampal neurons reduce the pool of new neurons available to support new memory encoding, and likely, in part, account for post-treatment memory disruptions experienced by cancer survivors [80,81]. 

## 3. Current Methods for Assessing Chemotherapy-Related Memory Disruption

Much of what is known about CRCI and its underlying cellular and molecular mechanisms has been identified in pre-clinical studies of rodents [22,31,72,82]. Pre-clinical models are critical for identifying physiological changes in response to various chemotherapy drugs with a high degree of cellular specificity [70,72], and have the advantage of controlling for confounding factors in human studies into the effects of chemotherapy drugs on neurocognitive function. These confounding factors include variations in drug types, dosage and treatment schedules, duration since treatment, methods of cognitive evaluation, as well as comorbidities and other forms of treatment. A major limitation to pre-clinical studies of cognition and behaviour as a model for CRCI, however, is that they fail to capture the nuanced cognitive disturbances experienced by cancer survivors. For example, while breast cancer survivors experiencing CRCI exhibit memory difficulties in various forms, standard tasks used to assess long-term memory in pre-clinical models are unidimensional (e.g., delayed place and object recognition tasks), and do not capture the complexities of human long-term memory processing. 

This limitation in test complexity is not unique to pre-clinical measures. The most common neurocognitive tests of long-term memory performance in breast cancer survivors measure verbal and visual memory using standardized list learning, word or object recognition, or free recall tasks following a delay. These are well established tasks that are sensitive to mild cognitive impairment associated with hippocampal impairment [83,84,85]. Given that encoding and retrieval of verbal and visual memory strongly engage left and right hemispheric regions (respectively), including hippocampus [86,87] using tests that are sensitive to detecting deficient hippocampal processing in breast cancer survivors provides a useful diagnostic indicator of basic memory dysfunction. Several longitudinal assessments of patients’ verbal and visual memory using the **California Verbal Learning Test** and the **Brief Visuospatial Memory Test-Revised**, for example, have identified lower scores relative to pre-treatment baseline and to healthy controls, persisting up to one year post-chemotherapy [88,89,90]. See Table 1, Table 2 and Table 3 for test details, and review of verbal and visual memory assessments in breast cancer survivors.

Other neurocognitive assessments of **episodic memory** employ **paired associates** learning tasks at encoding, requiring participants to later recognize paired items, words, or spatial contexts, presented during encoding and again during a recognition test [17,91]. Episodic memory involves recollection of details related to the ‘what, where, and when’ of unique events [92,93,94]. Using this method of assessment, reduced recognition memory for face-context pairings was observed in chemotherapy treated (Ch+) breast cancer survivors ten years following treatment, indicative of long-lasting memory interference [17]. See ‘Neuropsychological Tests (NPT)’ column in Table 1, Table 2 and Table 3 for assessment details. While these verbal and visual memory tasks are well established and validated memory assessments that can provide insight into potential deficits within the episodic memory domain, they are not reflective of the type of complex **declarative memory** processing required to support the encoding (formation) and recollection of real-life, everyday events and the related **semantic** information that is an intricate part of human memory for personal experiences [95].

### 3.1. Complex Declarative Memory Processing and CRCI

Declarative memory, or memory that can be voluntarily called into consciousness, is comprised of both episodic and semantic elements [93]. Semantic memory involves retrieval of facts or general knowledge that is not tied to a specific event, whereas episodic memory involves recollection of details for unique events [92,93,94]. Both encoding and retrieval of an episodic memory rely heavily on hippocampal engagement. Patients with damage to medial temporal lobe (MTL) structures including the hippocampus are disproportionately impaired in recalling the episodic components of previously experienced event memories, and will instead provide semantic elements related to the memory [93,96,97,98,99,100]. For example, if prompted to recall a story about a day at their job, an MTL patient could report facts about the company, their position within the company, and their boss’ name (preserved semantic memory retrieval), but would be unable to recall a specific event that occurred while working with their boss (impaired episodic memory retrieval).

Even in healthy individuals, the precise episodic elements of a memory are susceptible to forgetting over time whereas the semantic elements of a memory tend to be more stable [93,101,102]. For example, a healthy individual can likely recall their experience of yesterday’s staff meeting in vivid detail, including their position in the room, the attendance and appearance of their colleagues, the objects in the meeting room, the order in which their colleagues spoke, and specific phrases (episodic details for the event). If asked to recall a staff meeting from three years ago, that person is likely to remember few episodic details about the meeting, while recalling general, **schematic** features of the event (i.e., “It was in the conference room. Our boss sat at the front of the table. Each director gave their report”), and semantic information related to the event (i.e., “We have staff meetings every Wednesday at 3:30 PM. The conference room is on the 3rd floor)”. Despite this normal loss of memory for episodic details, if given a salient cue at the time of retrieval, healthy individuals can probably access those precise episodic details even after a very long time (i.e., “That was the meeting when Mikki brought pastries from Wisconsin. There were four large, round pastries on the conference table. They tasted very sweet.”).

**Table 1:** Summary of reports identifying chemotherapy-induced structural differences in the temporal lobes using MRI and associated memory disruption in breast cancer survivors.

References	Sample	Age	Tumor Stage	Menopausal Status	Treatment	Timepoints	Neuropsychological Tests	NPT Results	Imaging Results
Inagaki et al. [103]	Ch + MDE (*n* = 17), Ch+ (*n* = 51)	18–55	0–III	Post.M. (*n* = 10 Ch + MDE, *n* = 27 Ch+)	Chemo, ET, surgery	6 mo postsurgery (t1)	WMS-R: immediate and delayed verbal and visual memory tasks	=verbal and visual memory for both groups	=left and right HPC volume for Ch + MDE and Ch+
Yoshikawa et al. [104]	Ch+ (*n* = 44), Ch− (*n* = 31)	~48	0–I	Post.M. (*n* = 27 Ch+, *n* = 8 Ch−)	Chemo (CMF, AC, CAF, CPP, MF, 5FU, HCFU, or doifluridine), ET, RT, surgery	~3.5 yr postchemo (t1)	WMS-R: immediate and delayed verbal and visual memory tasks	=verbal and visual memory for both groups	=HPC volume between Ch+ and Ch− and between different chemotherapy regimens
Ferguson et al. [105]	Ch+ (*n* = 1), HC (*n* = 1) mono zygotic twins	60	II	-	Chemo (TAC), ET	22 mo postchemo (t1)	verbal memory: CVLT, Craft stories	=verbal memory for both twins	↑ WM lesion volumes and hyperintensities for Ch+ than HC
Inagaki et al. [106]	Ch+, Ch−, HC (*n* = 51–55/ group) at t1	18–55	0–I	Post.M. (*n* = 40 Ch+, *n* = 20 Ch−, *n* = 16 HC)	Chemo (AC, CMF, EC, PTX, 5FU, 5′-DFUR, HCFU, or UFT), ET, RT	1 yr postsurgery (t1) and 2 yr after t1 (t2)	WMS-R: immediate and delayed verbal and visual memory	-	↓ GM and ↓ WM in paraHPC, prefrontal, precuneus at t1 for Ch+ than Ch−; = GM and WM at t2
McDonald et al. [107]	Ch+ (*n* = 17), Ch− (*n* = 12), HC (*n* = 18)	~50	0–III	-	Chemo (CPP + DOX, ACT, or AC), ET, surgery	Baseline (t1), 1 mo (t2) and 1 yr postchemo (t3)	-	-	= GM at t1; ↓ GM bilateral paraHPC, STG at t2 than t1 and MTL at t3 than t1 for Ch+ than HC
Koppelmans et al. [13]	Ch+ (*n* = 177), HC (*n* = 368)	50–80	-	-	Chemo (CMF)	~21 yr postchemo (t1)	-	-	↓ GM, ↓ TBV, =WM, =left HPC volume for Ch+ and HC
Conroy et al. [108]	Ch+ (*n* = 24), HC (*n* = 34)	49–71	I–III	-	Chemo (AC, ACT, CAF, AT, CMF, CMF + CAF, taxane, ACT+ CAPE, or taxane + CAPE)	~6.4 yr postchemo (t1)	verbal memory: RAVLT, story recall, BLT	↓verbal memory for Ch+ than HC	↓ GMD in left temporal lobe for Ch+ than HC.
Kesler et al. [109]	Ch+ (*n* = 42), HC (*n* = 35)	~55	I–III	Post.M. (*n* = 33 Ch+, *n* = 18 HC)	Chemo (DOX + CPP, DOX + PTX, CPP + 5FU + PTX, or CPP + 5FU+ MTX), ET, RT	~5 yr postchemo	verbal memory: HVLT	↓ HVLT delayed recall for Ch+ than HC	↓ bilateral HPC volume for Ch+ than HC
Lepage et al. [110]	Ch+ (*n* = 19), HC (*n* = 19)	~50	I–III	Menstruating, peri.m, post.m (*n* = 2–9/ group)	Chemo (FECD, CD, or CPP + DOX), surgery	baseline (t1), 20 days (t2) and 1.5 yr postchemo (t3)	verbal memory: HVLT, CNS-VS-Verbal Memory Index; visual memory: BVMT-R, CNS-VS-Visual Memory Index	↓ NPT scores over time (non- significant) for both groups	↓ GM volume in temporal regions from t1 to t2 for Ch+ compared to HC, = GM at t3 between both groups
Apple et al. [73]	Ch+ (*n* = 16), HC (*n* = 18)	18–45	I-IV	Pre.M.	Chemo (ACT), ET	6–18 mo postchemo (t1)	episodic memory: Picture Sequence Memory Test	↓episodic memory for Ch+ than HC	↑inward deformation in bilateral HPC, ↓HPC volume for Ch+ than HC

Abbreviations: ↑, increase/higher scores; ~, approximately; ↓, reduction/lower scores; = no difference between groups; ≠, not the same/different scores; -, negative correlation; 5FU, fluorouracil; 5′-DFUR, doxifluridine; AC, CPP + DOX; ACT, DOX + CPP + taxane; AT, DOX + taxane; BLT, Brown Learning Test; BVMT-R, Brief Visuospatial Memory Test-Revised; CAF DOX + CPP + 5FU; CAPE, capecitabine; CD, CPP + DTX; Ch−, breast cancer patients that did not take chemotherapy; Ch+, breast cancer patients that took chemotherapy; chemo, chemotherapy treatment; CMF, CPP + MTX + 5FU; CNS-VS, computerized neurocognitive assessment vital signs; CPP, cyclophosphamide; CVLT, California Verbal Learning Test; DOX, doxorubicin; DTX, docetaxel; EC, epirubicin and cyclophosphamide; ET, endocrine therapy; FEC-D, 5FU + CPP + epirubicin + DTX; GM, gray matter; GMD, gray matter density; HC, healthy controls; HPC, hippocampus; HCFU, carmofur; HVLT, Hopkins Verbal Learning Test; MDE, major depressive episode; MF, MTX + 5FU; MTX, methotrexate; mo, months; n, sample size; NPT, Neuropsychological Tests; ParaHPC, parahippocampal; Peri.M., perimenopausal; postchemo, postchemotherapy treatment; Post.M., postmenopausal; Pre.M., premenopausal; PTX, paclitaxel; RAVLT, Rey Auditory Verbal Learning Test; RT, radiation therapy; STG, superior temporal gyrus; t1, testing session 1; t2, testing session 2; t3, testing session 3; TAC, CPP + DOX + docetaxel; TBV, total brain volume; UFT, tegafur/uracil; WMS-R, Wechsler Memory Scale-Revised; WM, white matter; yr, year(s).

**Table 2:** Summary of reports identifying chemotherapy-induced functional differences in the temporal lobes and memory disruption in breast cancer survivors using task-based fMRI.

References	Sample	Age	Tumor Stage	Menopausal Status	Treatment	Timepoints	Neuropsychological Tests	NPT Results	Imaging Results
Kesler et al. [111]	Ch+ (*n* = 14), HC (*n* = 14)	40–65	metastatic (*n* = 8), locally advanced (*n* = 6)	-	Chemo (CMF, or ACT), ET, RT	>6 mo postchemo (t1)	Verbal declarative memory encoding and recall task in fMRI	=verbal declarative memory for both groups	↑ right STG activation extending into paraHPC and left HPC during the verbal declarative encoding and recall task for Ch+ than HC
de Ruiter et al. [17]	Ch+ (*n* = 19), Ch− (*n* = 15)	~57	I-III	-	Chemo (FEC, or CTC), ET, RT, and surgery	~2 (t1) and 9 yr postchemo (t2)	verbal memory: CVLT; visual memory: WMS-R visual reproduction test; episodic memory: PA in fMRI	t1 to t2: ↓ PA for Ch+ than Ch−; ↓ visual and verbal memory for Ch+ than HC	↓ right PHG and MTG activation during PA task for Ch+ than Ch−
Lopez-Zuinini et al. [112]	Ch+ (*n* = 21), HC (*n* = 21)	31–64	I-III	Peri.M. (*n* = 2–4/group), Post.M. (*n* = 9–10/group)	Chemo (CPP + DOX, CPP + DTX, 5FU + CPP+ DTX + hepirubicin+ epirubicin or 5FU, TEC), surgery	baseline (t1), and 1 mo postchemo (t2)	verbal memory: verbal word list learning in fMRI	=verbal word learning for both groups	↓ activation in right STG, bilateral insula, and left inferior orbitofrontal gyrus during the verbal list learning task for Ch+ than HC
Apple et al. [113]	Ch+ (*n* = 16), HC (*n* = 18)	18–45	-	Pre.M.	Chemo, ET	~18 mo postchemo (t1)	episodic memory: Picture Sequence Memory Test and RWCR in fMRI	↓ episodic memory for Ch+ BCP than HC	↑ HPC FC in the left cuneus, lingual, precuneus, and right middle frontal gyrus during RWCR for Ch+ than HC

Abbreviations: ↑, increase/higher scores; ~, approximately; ↓, reduction/lower scores; = no difference between groups; ≠, not equal/different scores; +, positive; 5FU, fluorouracil; AC, CPP + DOX; ACT, DOX + CPP + taxane; AT, DOX + taxane; BCP, breast cancer patients; CAF, 5FU + CPP + DOX; CAPE, capecitabine; Ch−, breast cancer patients that didn’t take chemotherapy; Ch+, breast cancer patients that took chemotherapy; Chemo, chemotherapy treatment; CMF, CPP + MTX + 5FU; CPP, cyclophosphamide; CTC, CPP + thiotepa + carboplatin; CVLT, California Verbal Learning Test; DOX, doxorubicin; DTX, docetaxel; EBPM, event-based prospective memory; ET, endocrine therapy; FC, functional connectivity; FEC, 5FU + epirubicin + CPP; FFA, fusiform area; fMRI, functional magnetic resonance imaging; HC, healthy controls; HPCn, hippocampal network; mo, months; MTG, medial temporal gyrus; n, sample size; PA, paired associates; paraHPC, parahippocampal; Peri.M., perimenopausal; PFC, prefrontal cortex; PHG, parahippocampal gyrus; postchemo, postchemotherapy treatment; Post.M., postmenopausal; Pre.M., premenopausal; pTG, posterior temporal gyrus; PTX, paclitaxel; RT, radiation therapy; RWCR, novel recognition without cued recall; STG, superior temporal gyrus; t1, Testing session 1; t2, testing session 2; TBPM, time-based prospective memory; TEC, DTX + CPP + epirubicin; TG, temporal gyrus, WMS-R, Wechsler Memory Scale-Revised; yr, year(s).

**Table 3:** Summary of reports identifying chemotherapy-induced functional differences in the temporal lobes during resting state fMRI in breast cancer survivors.

References	Sample	Age	Tumor Stage	Menopausal Status	Treatment	Timepoints	NPT Tests	NPT Results	Imaging Results
Bruno et al. [114]	Ch+ (*n* = 34), HC (*n* = 27)	40–74	I-IV	Post and Pre M.	Chemo (ADM + CPP + PTX, CPP + MTX + 5FU and ADM + CPP or, CPP + MTX + 5FU), ET, RT	~5 yr post-treatment (t1)	verbal memory: HVLT	=HVLT immediate, ↓HVLT delayed for Ch+ than HC	↓ global and regional network measures in bilateral STG for Ch+ than HC; ↑network hubs in bilateral STG and left HPC for HC than Ch+
Tao et al. [115]	Ch+ (*n* = 33), HC (*n* = 31)	26–52	I-III	-	Chemo (DOX, CPP, PTX), surgery	-	-	-	↓ FC in the DMN for Ch+ compared to HC
Cheng et al. [116]	Ch+ (*n* = 34), HC (*n* = 31)	~50	-	-	Chemo (DOX, 5FU, CPP, or PTX)	-	prospective memory: EBPM, TBPM	↓ EBPM, TBPM for Ch+ than HC; =scores between HC and Ch−	↑ FC between HPC seed and bilateral vmPFC, dlPFC, inferior and superior parietal lobules, pCC, and precuneus for Ch+ than HC
Chen et al. [117]	Ch+ (*n* = 16), HC (*n* = 14)	>60	I-III	-	Chemo (TC or other), surgery	baseline (t1), 1 mo postchemo (t2)	episodic memory: Picture Sequence Memory Test	=NPT scores for Ch+ and HC across t1 and t2	↑ ALFF from t1 to t2 in a single cluster including bilateral subcallosal gyri and right anterior cingulate gyrus for Ch+ compared to HC; =rs-fMRI from t1 to t2 for Ch+ and HC
Feng et al. [118]	Ch+ (*n* = 29), HC (*n* = 25)	30–50	I-III	Pre.M.(*n* = 17–20/group), menopausal (*n* = 8–9/group)	Chemo (ACT, TEC), surgery	baseline (t1), 1 week postchemo (t2)	verbal memory: AVLT	↓ AVLT from t1 to t2 for Ch+ than HC	↑ FC between left anterior HPC and left MTG and STG, and between the right posterior HPC and left STG for Ch+ compared to HC
Feng et al. [119]	Ch+ (*n* = 7), HC (*n* = 19)	35–55	I-III	Pre.M. (*n* = 11/group), menopausal (*n* = 6–8/group)	Chemo (ACT, TEC), ET	baseline (t1), 1 week (t2) and 6 mo postchemo (t3)	verbal memory: WDT	↓ WDT from t1 to t3 for Ch+ than HC	↓ FC in ADMN, PDMN, LFPN, RFPN, SRN, CN from t1 to t3 for Ch+ than HC

Abbreviations: ↑, increase/higher scores; ~, approximately; ↓, reduction/lower scores; = no difference between groups; ≠, not the same/different scores; >, above; 5FU, Fluorouracil; ACT, DOX + CPP + taxane; ADM, adroamycin; ADMN, anterior default mode network; ALFF, amplitude of low-frequency fluctuation; AVLT, Auditory Verbal Learning Test; BCP, breast cancer patients; Ch−, breast cancer patients that didn’t take chemotherapy; Ch+, breast cancer patients that took chemotherapy; chemo, chemotherapy treatment; CN, Central network; CPP, cyclophosphamide; dlPFC, dorsolateral prefrontal cortex; DMN, default mode network; DOX, doxorubicin; DTX, docetaxel; ET, endocrine therapy; FC, functional connectivity; HC, healthy controls; HVLT, Hopkins Verbal Learning Test; HPC, hippocampus; ITG, inferior temporal gyrus; LFPN, left frontoparietal network; mo, months; MTG, middle temporal gyrus; MTX, methotrexate; n, sample size; NPT, Neuropsychological Test; ParaHPC, parahippocampal; pCC, posterior cingulate cortex; PDMN, posterior default mode network; postchemo, postchemotherapy treatment; Post.M., postmenopausal; Pre.M., premenopausal; pSTG, temporal pole of superior temporal gyrus; PTX, paclitaxel; ReHo, regional homogeneity; RFPN, right frontoparietal network; rs-fMRI, resting state functional magnetic resonance imaging; RT, radiation therapy; SRN, Self-referential network; STG, superior temporal gyrus; t1, Testing session 1; t2, testing session 2; t3, testing session 3; TAC, DTX + ADM + CPP; TC, DTX + CP; TEC, DTX + CPP + Epirubicin; VN, visual network; WDT, Auditory verbal learning memory; yr, year(s).

Observations of differential loss of memory details can be accounted for by the **Trace Transformation Theory**, which posits that episodic memories are consolidated in rich contextual and perceptual detail within the hippocampus. So long as the memory remains accessible, the hippocampus continues to be required for the retrieval of contextually and perceptually detailed elements of the memory. Over time, episodic memories are transformed into less detailed schematic memories that capture the essential features or gist of the original event. Storage and retrieval of this form of the memory are supported by neocortical regions, with the prefrontal cortex playing a particularly important role. The detailed hippocampus-dependent version, and the transformed schematic version of the memory, can co-exist in the healthy brain, and the available cues at the time of retrieval will direct which version is retrieved [92,95,120]. Regardless of the age of the memory (recent, i.e., yesterday’s staff meeting; remote, i.e., staff meeting from three years ago), the hippocampus continues to be engaged when retrieving episodically detailed elements of event memories [120,121,122].

Given this profile of memory consolidation, it is probable that even subtle hippocampal disruption, such as that resulting from chemotherapy-induced neurogenic suppression or hippocampal atrophy would result in selective impairment in the retrieval of episodic memories. Such results have been identified in preclinical rodent models in which adult hippocampal neurogenesis has been ablated using chemotherapy [60,61] or cranial radiation [39,60,123,124], resulting in impaired context memory which is a measure of episodic-like memory in rodent memory models.

### 3.2. Autobiographical Memory and CRCI

**Autobiographical memory** is a unique form of declarative memory that unfolds over time, involves temporal and spatial sequencing of an event, and is comprised of a complex interaction of episodic and semantic elements of a personally experienced event. To recall an autobiographical memory, details that must be accessed from memory stores, reconstructed and elaborated upon during the retrieval process [125,126]. Patients with MTL damage exhibit relative preservation of older autobiographical memories experienced long before hippocampal damage, and a temporal gradient, with memories experienced more recently prior to hippocampal insult being more susceptible to disruption. The remote **retrograde** memories that are preserved in the presence of hippocampal damage, however, tend to lack episodic specificity, and rather retain a more gist-like and semantic version of the event [100,127,128]. 

Despite the pervasive reports of memory impairment in those experiencing CRCI and the known susceptibility of the hippocampus to the neurotoxic effects of chemotherapy, only a limited number of studies have investigated autobiographical memory processing in breast cancer survivors. One early investigation using the Autobiographical Memory Task found that Ch+ breast cancer survivors demonstrated reduced ability to produce ‘specific’ autobiographical memories in response to positive, negative, or neutrally valenced cue words when compared with heathy controls [129]. Rather than retrieving episodic details, Ch+ breast cancer survivors produced overgeneralized memories, thought to result from an impairment in the generative retrieval process that does not reach the elaboration phase required for event-specific memory retrieval [126,130]. The Autobiographical Memory Task [131] used here, however, provides limited insight into potential temporal differences in memory retrieval of personal event memories, as it does not take into account the age of the retrieved memories (recently experienced post-treatment events vs. remotely experienced pre-treatment events), nor does it account for the qualitative content of the retrieved memory beyond classifying it as ‘specific’, ‘general’, or a ‘non-memory’. This is an important consideration, as impairment resulting from chemotherapy-induced hippocampal neurotoxicity may differentially impair the more specific, episodic components of the recalled event while leaving the more general schematic and semantic components unaffected [105].

Another study assessed both pre- and post-treatment autobiographical memories in breast cancer survivors using the more rigorous **TEMPau task**, a semi-structured interview that assesses memories for unique events occurring at specific times and places within three lifetime periods: the five years before treatment, the cancer treatment period, and the 12 month post-treatment period [132]. The results identified an overall reduction in autobiographical memory retrieval ability in Ch+ breast cancer survivors, with a specific deficit in retrieval of temporal details. Given the observed deficit in temporal memory processing, it is unfortunate that the study failed to assess potential differences in temporally graded retrograde memory to determine if the more remote (five year old) memories were less impaired than the more recently experienced autobiographical memories. As the process of memory transformation and retrieval network reorganization occurs over time, even in healthy individuals [92,95,120,121,133,134], it is plausible that deficits in episodic memory retrieval for complex event memories in Ch+ individuals will be less evident for more remote memories given the natural forgetting of episodic memory details over time, and the reduced reliance on the hippocampus for this type of memory.

Several tests have been developed to probe the qualitative content of autobiographical memories, including the Autobiographical Memory Interview [135] and the **Autobiographical Interview** [136]. The Autobiographical Interview is a structured memory interview that distinguishes between retrieved episodic details that are unique to the retrieved experience (internal details, i.e., “I was wearing a blue bathing suit and swimming in the cold lake with my brother when he came to visit me last weekend”) and semantic aspects of memory (external details, i.e., “It was my favorite bathing suit, I’m a great swimmer, and we used to go to the lake every summer”). The Autobiographical Interview allows for the classification of sub-categories of internal details to identify domains of episodic memory that are susceptible to impairment with a high degree of specificity (perceptual, emotion/thought, time, place, event details). 

**Figure 1:**
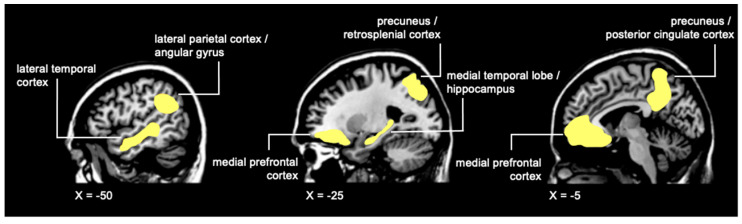
Regions commonly activated during memory recollection, comprising the recollection networks.

The Autobiographical Interview has been used to identify episodic memory disturbances in many patient populations involving medial-temporal lobe disruption [128,137,138,139], and in normal aging [140,141], but has not been used to assess complex event memory processing abilities in chemotherapy-treated breast cancer survivors. The Autobiographical Interview is a powerful tool for detecting subtle episodic memory deficits in the presence of even minor hippocampal dysfunction. A study using the Autobiographical Interview [136] in pediatric brain tumor patients found that chemotherapy and craniospinal radiation were associated with significant impairment in patients’ ability to recall specific from personal episodic events experienced following treatment, whereas their ability to retrieve general semantic details from the same events was unimpaired compared to healthy controls. Interestingly, the quality of details recalled for remote, pre-treatment memories was unimpaired, suggesting that, as with MTL patients, chemotherapy and cranial radiation treatments selectively impair the ability to form new, highly detailed autobiographical memories, while leaving previously established memories unaffected. This pattern of impairment was accompanied by reduced overall volume in the hippocampus, as well as the fornix, the main efferent white matter tract projecting from the hippocampus to the mammillary bodies in the diencephalon [142]. A moderate reduction in volume was also observed in the precuneus, but other nodes of the **recollection network** (Figure 1), including the medial prefrontal cortex (mPFC), were unaffected. This may account for the preservation of remote retrograde memories, which reorganize and recruit prefrontal cortical regions as the memories age and become less episodically detailed over time [92,95,142,143].

To date, the few assessments of autobiographical memory performance in breast cancer survivors have identified autobiographical memory as a cognitive domain that is vulnerable to the effects of chemotherapy, yet the measures used to assess the qualitative content of patients’ memory have lacked the rigor to objectively assess the subdomains of episodic memory retrieval [106,132]. Critically, they have not accounted for the differential effects of chemotherapy-mediated disruptions along a temporal gradient, including of retrograde, **anterograde**, and future imagining of autobiographical events. Further, identifying structural and functional differences or changes in the hippocampus and throughout the recollection network that may be mediating autobiographical memory dysfunction will be essential in identifying therapeutic targets. These are important considerations moving forward, given the susceptibility hippocampal-dependent memory processing to chemotherapy treatments, and its implications for maintaining quality of life in cancer survivors. 

## 4. Neuroimaging Assessments

### 4.1. Chemotherapy-Induced Structural Changes to Hippocampus and the Temporal Lobes

Within the MTL, and hippocampi specifically, notable structural and functional differences have been observed in breast cancer patients following chemotherapy treatment (Table 1), corroborating findings from pre-clinical studies in rodents [27,49,69]. Early investigations first considered the influence of post-traumatic stress [144], and post-treatment depressive episodes [103] on hippocampal volume in breast cancer survivors. In a series of MRI studies of Japanese breast cancer survivors conducted three years post-treatment (see Table 1 for patient demographic and chemotherapy treatment details), no differences in left or right hippocampal volume, nor overall brain volume, were observed in Ch+ breast cancer survivors who had experienced a first depressive episode following breast cancer treatment [103]. In the same sample, survivors who reported experiencing distressing and intrusive cancer-related recollections for at least one month during the post-treatment interval had marginally smaller left hippocampal volume, relative to survivors with no history of distressing recollections [144]. The quality or content of these recollections was not probed, limiting any conclusions that could be drawn related to the episodic memory performance of these breast cancer survivors. Despite slightly smaller hippocampal volume, standardized tests of delayed verbal or visual memory performance using the **Wechsler Memory Scale-Revised** suggest that the occurrence of these distressing recollections did not impair general memory processing. 

Secondary analyses of these data, including the addition of a sample of non-chemotherapy-treated (Ch−) breast cancer survivors, failed to find any difference in hippocampal volume, whole brain volume, or any differences in delayed verbal of visual memory performance between Ch+ and Ch− survivors [104]. Given that scans and cognitive assessment were performed three years following treatment, the study suggested that a longitudinal approach including earlier timepoints may be required to detect potential chemotherapy-induced impairment in hippocampal volume and morphology.

Accordingly, structural imaging of both Ch+ and Ch− breast cancer survivors approximately one year following surgery and chemotherapy treatment identified smaller gray matter and white matter volumes within the parahippocampus, adjacent to the hippocampus, and recollection network regions including the prefrontal cortex and precuneus in Ch+ survivors [106]. Smaller gray matter volume within these regions was not evident three years post-treatment [106], consistent with their earlier null findings in a sample of survivors after three years [104]. See Table 1 for a summary of MRI studies finding chemotherapy-related disruptions in the hippocampus and temporal lobes. 

Since these early studies highlighting the need for a more longitudinal approach to monitoring chemotherapy-induced neural alterations, many subsequent MRI studies of brain volume and morphology have identified structural differences in white matter tracts and gray matter volumes across various brain regions in Ch+ breast cancer survivors [13,17,105,109,145,146]. 

**Figure 2:**
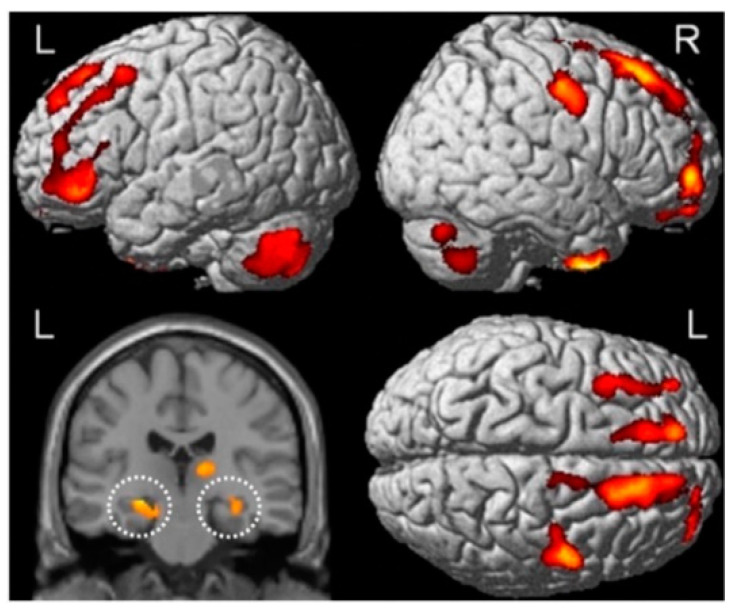
Voxel-based morphometry identified gray matter density declines (warm colours) between pre-treatment baseline and 1-month post-chemotherapy, notably within bilateral hippocampal and parahippocampal regions (white dashed circles). Abbreviations: R, right hemisphere; L, left hemisphere. Adapted from McDonald et al. [107].

The first MRI study to track longitudinal changes in gray matter volume across the whole brain using **voxel-based morphometry (VBM)** prior to, and following, chemotherapy treatment found significantly lower gray matter volumes within the bilateral hippocampus, parahippocampus, superior temporal gyrus, and regions in the frontal lobes, cerebellum, and thalamus just one month following treatment (Figure 2). Widespread density reductions were largely transient, with recovery of gray matter volume observed in the superior temporal regions one year following treatment in these same patients, though reduced density within the MTL and frontal lobes largely persisted after one year [107]. Gray matter density reductions were not observed in Ch− breast cancer survivors, suggesting that the persistent gray matter density deficits were not the result of cancer-related disturbances, but rather due to chemotherapy-induced neurotoxicity. A follow-up study incorporating fMRI performed 3–10 years post-treatment, confirmed lower gray matter densities within the left temporal lobe, and hypoactivation within the left middle temporal gyrus, while performing a working memory n-back task. No functional assessments with long-term memory tasks were performed, but the authors report impaired delayed memory scores on the **Rey Auditory Verbal Learning Test** in Ch+ breast cancer survivors. This memory deficit may be mediated, in part, by the observed left temporal lobe structural and functional disruption in Ch+ survivors [108].

A subsequent longitudinal study using VBM to assess gray matter volume changes one month, and one year post-treatment identified structural changes in the temporal lobes relative to pre-treatment baseline measures. A significant decline of volume in right hippocampus and right superior and middle temporal gyri was evident as early as one month following chemotherapy, and persisted one year post-treatment. Surprisingly, verbal and visual memory showed only modest impairment over time, despite the notable declines in temporal lobe gray matter [110].

A sample of Ch+ breast cancer survivors imaged between 1 and 12 years post-treatment revealed persistent effects of treatment on hippocampal volumes, with smaller left hippocampal volumes and inferior performance on the **Hopkins Verbal Learning Test** memory task relative to controls. In a sub-set of sampled patients, smaller left hippocampal volumes were associated with increased circulating pro-inflammatory cytokine expression of IL-6 and TNF-α [109]. While speculative, the cause of volume reductions within the detectable by MRI are likely associated with underlying cellular and molecular disturbances targeting the hippocampus, including suppressed neurogenesis, and dendritic atrophy [26,28].

### 4.2. Functional Specialization along the Hippocampal Long-Axis and Implications for Memory Performance following Chemotherapy

Chemotherapy-treated women within 18 months of completing treatment had significantly altered hippocampal morphology, with bilateral inward deformation predominantly within the anterior portion of the hippocampal long-axis, and smaller overall hippocampal volume. This deformity was associated with poorer episodic memory performance on the **Picture Sequence Memory Task** and with self-reported cognitive difficulties [73]. 

Hippocampal deformity within the anterior region may differentially impact memory processing, as the hippocampus is functionally specialized along its long axis and has unique structural connectivity in its anterior and posterior regions. The anterior hippocampus (analogous the ventral hippocampus in rodents) has connections with the ventromedial prefrontal cortex (vmPFC), and is associated with the processing of schematic memories [92,147]. The posterior hippocampus (analogous to dorsal hippocampus in rodents) [148] is thought to be involved in processing more fine-grained details that characterize vivid and perceptually detailed episodic memories [92,147].

In their study of autobiographical memory in breast cancer survivors 18 months post-treatment, Bergouignan and colleagues (2011) observed a specific deficit in recalling temporal details within autobiographical memory [132]. They also found reduced posterior hippocampal volume, which likely underlies the deficit in episodic memory retrieval, given the putative role of posterior hippocampus in processing fine-grained spatio-temporal aspects of episodic memory [147,149,150]. The posterior hippocampus has also been shown to be activated during autobiographical memory elaboration which requires the production of perceptually detailed elements of the memory [126], while connectivity between anterior hippocampus and vmPFC regions is more strongly engaged during the initial general construction phase of autobiographical memory retrieval [125,126,151,152]. These regional specializations in autobiographical memory processing may also account for the overgeneralized autobiographical memories reported by Bergouignan et al. [132], in the case of posterior hippocampal atrophy or shrinkage. Taken together, these findings suggest that chemotherapy-induced regional disruptions within the hippocampus may be indicative of the types of memory dysfunction a patient is likely to develop.

### 4.3. Chemotherapy-Induced Functional Disruptions in the Temporal Lobes and Broader Recollection Network

**Figure 3:**
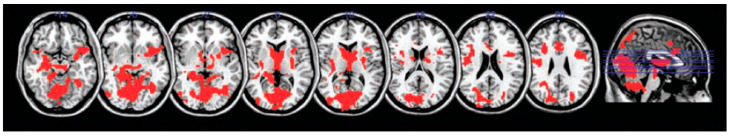
SPM analyses identified hyperactivity (warm colours) during accurate delayed verbal memory recognition in Ch+ breast cancer survivors compared to healthy control. Adapted from Kesler et al. [111].

An early study of fMRI neural dynamics and memory performance identified significantly greater activity across broad regions of the recollection network (Figure 1) in Ch+ breast cancer survivors performing a delayed verbal memory recognition task. Hyperactivity was observed in left hippocampus, bilateral parahippocampus gyri, right superior temporal gyrus, bilateral precuneus, right cingulate gyrus, and throughout several regions of the frontal lobes (Figure 3) [111]. The recognition accuracy of Ch+ breast cancer survivors was comparable to healthy controls. These results suggest that successful memory processing following chemotherapy is supported by compensatory over-recruitment of key nodes of the temporal lobes and the recollections network, reflective of inefficient neural processing. See Table 2 for a summary of fMRI studies showing chemotherapy-related disruptions in the hippocampus and temporal lobes during performance of memory tasks.

While network hyperactivity may reflect a compensatory response supporting memory performance following chemotherapy treatment, network hypoactivity has been associated with poor memory performance. A study of fMRI neural dynamics in Ch+ and Ch− breast cancer survivors conducted ten years after the completion of a high-dosage chemotherapy treatment found long-lasting hypoactivation of the parahippocampal gyrus in Ch+ survivors during encoding of a paired associates episodic memory task in which participants were shown a series of combinations of faces and contexts (i.e., a living room). During a subsequent recognition task in which participants had to judge the accuracy of the face-context pairings after a delay of several minutes, Ch+ breast cancer survivors had lower recognition accuracy scores than Ch− survivors [17]. This study provided support for the persistent altered neural dynamics within the MTL associated with chemotherapy treatment by accounting for cancer-related complications also experienced by the Ch− survivors. These data strongly suggest that altered neural dynamics with the MTL mediate the occurrence of episodic memory impairment, and can account for the high incidence of persistent memory loss in Ch+ breast cancer survivors. 

Subsequent longitudinal investigation using this same paired associates task in Ch+, Ch−, and healthy controls [91] identified no differences between groups during a pre-treatment baseline assessment, or during a 6-month post-treatment assessment on the face-context memory recognition task. While all groups showed robust activation of the hippocampus during the recognition task, no notable differences were seen between Ch+ and Ch− or healthy controls at the 6-month post-treatment period. When compared with their earlier findings [18] which find long-term disruption of parahippocampal processing ten years post-treatment, these findings suggest that identifying disruptions within the retrieval network in response to chemotherapy may develop over time. Their findings highlight the importance of tracking the development of neural dynamic disruptions longitudinally at repeated time points in patients to better understand the temporal profile of the development and persistence of CRCI.

Another study involving longitudinal tracking of functional networks in breast cancer survivors identified chemotherapy-induced changes in a widespread network of regions while performing a verbal memory task one month following chemotherapy [112]. Relative to patients’ pre-treatment baseline, network hypoactivity was observed in the right superior temporal gyrus, bilateral insula, and left inferior orbitofrontal gyrus, during recognition testing. Differences in functional network activity during the recognition memory task were also observed between Ch+ survivors and healthy controls, most notably in the superior and middle temporal gyrus, the left insula and superior temporal pole, and several frontal regions (Table 2). No deficits in verbal recognition memory performance were evident in Ch+ breast cancer survivors when compared to their baseline accuracy levels, or when compared to healthy control performance, despite network hypoactivity during the task. Thus, during a recognition task with relatively low cognitive demands, network hypoactivity did not result in detectable deficits in performance. Interestingly, in participants reporting high levels of fatigue, the hippocampus was more highly activated in Ch+ patients than controls, suggesting that successful recognition memory when highly fatigued requires extra engagement of hippocampal processing to support cognitive performance.

A preliminary study investigated eye-tracking during fMRI scanning in Ch+ patients and healthy controls while they performed the Picture Sequence Memory Test, an established spatial recognition memory task that is sensitive to hippocampal dysfunction [153]. While Ch+ patients were not impaired on the recognition task during scanning, they showed reduced eye-movement based discrimination, a measure of **implicit memory** (non-declarative memory) for the task. Reduced eye-movement discrimination was associated with hippocampal hypoactivation and smaller hippocampal volume, compared to control levels [154]. The connection between the observed implicit memory deficit and hippocampal abnormalities in Ch+ patients is not clear, as implicit memory is not considered to be dependent on the hippocampus [155,156,157]. In later task-based **functional connectivity** analyses of these data, Apple and colleagues (2018) identified strong intra-hippocampal connectivity for both Ch+ patients and controls, but Ch+ patients showed evidence of enhanced hippocampal connectivity with the left cuneus and precuneus, lingual gyrus, and right middle frontal gyrus compared to healthy control levels. Higher hippocampal connectivity with the precuneus was associated with higher reports of subjective cognitive concern scores in Ch+ patients, suggesting that hyper-connectivity within these nodes of the recollection network may be compensatory in supporting normal memory performance and needed to overcome anxiety-induced behavioural deficits associated with subjective concern over one’s cognitive abilities [113].

### 4.4. Recollection Network and Default Mode Network (DMN) Irregularities: Implications for Chemotherapy-Related Memory Impairments and Deficits in Episodic Future Thinking

Many of the neural regions identified as comprising the recollection network overlap with nodes of the **default mode network** (DMN). The DMN is a collection of brain regions that are active when engaged in passive, internally focused cognition (mind wandering) or during a resting state [158,159,160,161,162]. During rest or mind wandering, the brain engages in recollection and in future thinking (planning, imagining) [137,163]. Neuroimaging studies have shown that both future thinking and recollection engage the same core network of brain regions including the mPFC, lateral and medial temporal regions (hippocampus and parahippocampal cortex), and lateral and medial parietal regions (precuneus and retrosplenial cortex) (Figure 1), suggesting a similar underlying neural mechanisms mediating past and future memory processing [137,163,164,165,166,167]. This common DMN/recollection network engaged during mind wandering is an adaptive process that has been proposed to integrate and recombine associations from experiences stored in episodic memory to predict possible future situations in a process of ‘**constructive episodic simulation’** [137,168,169].

Prospective memory is a form of future thinking that involves planning and remembering to execute a task in the future [170]. It is mediated largely by regions within the frontal lobes (notably Brodmann Area 10) [171,172] and MTL [170,173], and is sensitive to disruption following chemotherapy [116,174,175]. **Episodic future thinking** is a complex form of prospective thinking which involves imagining or mentally projecting oneself into the future in order to pre-experience events. This process relies on similar cognitive processing and neural network activation involved in episodic recollection [176,177,178]. Given the overlap in functional activity for past and future episodic thinking, and observations of deficits in future imagining in individuals with MTL damage [128,135,178,179,180,181], it is plausible that deficits in episodically-detailed future thinking may occur following chemotherapy.

It is unsurprising that individuals with damage or dysfunction within the MTL, including key nodes of the recollection and DMN networks, engage in episodically impoverished mind-wandering. Mind wandering occurs in individuals with MTL disruption, but unlike healthy adults who report thoughts and recollections about the past, and future imagining during mind wandering, individuals with MTL damage report more semantically based thoughts about the present. This reflects an inability to engage hippocampally-mediated recollective processing required for episodic memory retrieval or episodic future thinking [182]. 

Bruno and colleagues [114] were the first to identify brain-wide resting state network irregularities in Ch+ breast cancer survivors. Their analysis of resting state activity identified lower global clustering scores in patients, which is indicative of inefficient neurotransmission between hub regions. Hubs are highly interconnected neural nodes that enable efficient network neurotransmission. They also identified several network hubs in the superior temporal gyrus, hippocampus and amygdala in controls that were not evident in breast cancer survivors during the resting state. Inefficient regional hub connectivity and global network processing may underlie reports of hyper-activity during cognitive task performance in cancer survivors, as the network must work harder to communicate across regions due to reduced direct connectivity [111,113]. The network connectivity inefficiencies identified at rest were associated with lower delayed verbal memory scores, and subjective reports of memory difficulties in this sample of Ch+ breast cancer survivors [114]. See Table 3 for a summary of resting state fMRI studies finding chemotherapy-related disruptions in the hippocampus and temporal lobes.

Modelling analyses by Kesler and colleagues (2017) found that pre-treatment resting state network dynamics can be used to predict the development of cognitive impairment in the first year following chemotherapy treatment, suggesting that irregularities in network dynamics are already detectable at the time of disease onset, and are further exacerbated by chemotherapy treatment [183]. Using **multi-voxel pattern analyses (MVPA)** of 19 seed regions within the DMN during a resting task, Kesler et al. (2013) were able to distinguish Ch+ breast cancer survivors from Ch−, from healthy controls with a high degree of accuracy [184]. MVPA is a neuroimaging technique that uses an individual’s pattern of neural activity during a task or during rest to predict their cognitive state or condition [185]. MVPA was unable to distinguish Ch− breast cancer patients from healthy controls above chance levels using these same regions of interest, suggesting that differences in DMN dynamics between groups was associated with chemotherapy treatment, and not due to the disease state itself.

Seed-based connectivity analyses during a resting state task by Cheng and colleagues [116] identified enhanced hippocampal functional connectivity between regions of the DMN including bilateral vmPFC and dlPFC, inferior and superior parietal lobules, pCC, and precuneus in Ch+ breast cancer survivors relative to healthy controls. They also identified prospective memory impairments associated with hippocampal hyper-connectivity in Ch+ breast cancer survivors relative to controls, and compared to pre-treatment prospective memory performance levels for both event and time-based tasks. Their findings suggest that this altered hippocampal connectivity with the rest of the DMN underlies prospective memory difficulties observed in this same sample of breast cancer survivors [116]. Similarly, post-treatment increases in resting state hippocampal connectivity was identified along the hippocampal long-axis relative to the pre-treatment connectivity pattern [118]. Long-axis connectivity changes during rest were associated with poorer auditory memory scores in Ch+ relative to controls. 

Post-treatment perturbations in DMN connectivity patterns have been proposed as a potential biomarker of chemotherapy-induced neurotoxicity, and assessment of patient’s resting state network dynamics may be a useful non-invasive diagnostic tool for identifying those requiring cognitive intervention post-treatment [186]. While this review focused on resting state network disruption involving the temporal lobes and its relation to the recollection network and memory processing, it should be noted that disrupted resting state network connectivity in Ch+ breast cancer survivors has also been widely reported using functional connectivity analyses of non-temporal nodes of the DMN, most notably within the frontal and parietal lobes, and accompanied by working memory and executive function impairments [115,116,119,187,188,189].

## 5. Other Contributing Psychosocial Factors Affecting Memory Performance

Fatigue, anxiety, stress, and other psychosocial factors likely influence cognitive performance, and confound interpretations of performance on standard neurocognitive tests [112,113,190]. Observations in breast cancer patients prior to chemotherapy reveal that disease onset alone is sufficient to induce several physiological changes which may account, in part, for observed cognitive impairments. These changes include disputations in functional network dynamics in frontal and parietal regions [183], and related impairments in executive function, working memory [183,191,192], response inhibition [191], and planning [91] in early-stage breast cancer patients.

Self-perceived impairments in memory are a common complaint following chemotherapy treatment [6,146,193]. Subjective accounts are an important indicator of an individual’s perceived cognitive abilities. However, for complex event memories, there is evidence that an individual’s confidence in the quality of their memory is not an especially reliable measure of its accuracy [194,195,196]. In this case, perceived memory difficulties may rather reflect other psychosocial behavioural conditions such as stress, depression, or anxiety [197,198].

Following a cancer diagnosis, approximately 14% of patients develop cancer-related post-traumatic stress disorder [199,200]. There is evidence that retrieval of autobiographical events surrounding the time of diagnosis is altered in recently diagnosed patients [201,202]. This alteration, or distortion, of self-related event memories also intrudes into episodic future thinking, with a bias towards negative affective details when thinking about the future. High levels of anxiety associated with a diagnosis have been found to impair the emotional content of autobiographical memory retrieval, even prior to the initiation of chemotherapeutic intervention, identifying autobiographical memory as a cognitive domain that is highly susceptible to distortion in breast cancer patients [203].

## 6. Recommendations and Conclusions

This review and commentary on the current state of the memory-related literature in the field of CRCI has identified a gap in our knowledge of the impact of chemotherapy on complex episodic memory processing and alterations to the recollection network. In light of the susceptibility of the hippocampus to chemotherapy-induced neurotoxicity, and the critical role of the hippocampus in episodic memory processing, it is surprising that there has been so little investigation of complex event and autobiographical memory processing in cases of CRCI. A multidisciplinary approach that combines complementary assessments of lab-based neurocognitive episodic memory performance, with more complex real-life event memory assessments (e.g., autobiographical memory) is needed to fully characterize the specific memory domains affected by cancer onset and chemotherapy treatments.

While chemotherapy-related suppression of neurogenesis is a leading candidate underlying the memory disruptions and hippocampal functional impairment, other physiological factors likely also contribute to these deficits, including white-matter degradation in the hippocampus and throughout other regions of the recollection network [145], pro-inflammatory cytokine [23,184] and microglial activation [26,27], among others. Multi-modal and longitudinal neuroimaging assessments are required to better capture structural and functional changes that develop and persist over time. These findings are essential to identifying the underlying mechanisms that contribute to cognitive impairments within the domains of complex event memory processing, and future thinking. The limited investigations to date highlight the need for systematic investigation of this cognitive domain, and further review of the medial-temporal lobe and recollection network alterations associated with CRCI-induced memory disturbances in cancer survivors.

Investigation of these cognitive domains in CRCI are still in their infancy. Given the importance of autobiographical memory to a person’s personal history, sense of identity, and ability to plan for the future, an under-appreciation of how this memory domain may be impaired by standard cancer treatments, has the effect of diminishing the negative experiences of breast cancer survivors, and neglecting cognitive problems that could benefit from intervention strategies.

## Abbreviations

CA1	cornu Ammonis 1
CA3	cornu Ammonis 3
Ch−	breast cancer patients that didn’t receive chemotherapy
Ch+	breast cancer patients that were treated with chemotherapy
CRCI	chemotherapy-related cognitive impairment
dlPFC	dorsolateral prefrontal cortex
DMN	default mode network
fMRI	functional magnetic resonance imaging
IL-1β	interleukin-1β
IL-6	Interleukin 6
mPFC	medial prefrontal cortex
MRI	magnetic resonance imaging
MTL	medial temporal lobe
MVPA	multi-voxel pattern analyses
pCC	posterior cingulate cortex
TNF-α	Tumor necrosis factor α
VBM	voxel-based morphometry
vmPFC	ventromedial prefrontal cortex

## Glossary

Anterograde memory: formation of new memories after a specific point in time.

Autobiographical memory: memory for personal experiences and facts about oneself.

Autobiographical Interview: a standardized, structured interview that asks participants to describe memories from personal events that occurred at a specific time and place (e.g., 5 years ago, 1 year ago, and 1 week ago). The reported narratives are scored to identify internal details that are unique to the event (episodic details—perceptual, emotion/thought, time, place, event details) and external details that are not unique to the event (semantic details).

Brief Visuospatial Memory Test-Revised: a standard neurocognitive task where participants are presented with several figures and asked to immediately draw the figures from memory, which is then repeated for three consecutive trials. After a 25-min delay, they are asked to draw the image from memory and complete a recognition task. This task measures visuospatial learning and memory abilities.

California Verbal Learning Test: a standard neurocognitive task where participants are presented with two lists of words and are asked to recall as many as they can remember using semantic categorization immediately after they are presented. Next, participants complete a recognition task after a 15-min delay. This task assesses encoding, storage, retrieval, and recognition of verbal memory.

Constructive episodic simulation: a cognitive process that integrates and recombines associations from experiences stored in episodic memory in order to predict possible future situations based on past experiences.

Declarative memory: a category of explicit long-term memory comprised of semantic and episodic memory, including memory for facts, general knowledge, and personally experienced events.

Default mode network: a network of brain regions engaged during passive, internally focused cognition (mind-wandering) or rest. Regions include the mPFC, parahippocampus, retrosplenial/posterior parietal cortex, precuneus, lateral parietal cortex/angular gyrus.

Episodic future thinking: imagining or mentally projecting oneself into the future to mentally pre-experience events.

Episodic memory: a component of declarative memory involving conscious recollection of an event occurring at a specific time and place. Episodic memory is characterized by a sense of mentally re-experiencing the contextual and perceptual details of a personal event or episode (i.e., visiting the State Fair of Texas last week with my friend Brian).

Functional connectivity: correlated activation of brain regions during a functional neuroimaging task.

Hopkins Verbal Learning Test: a standard neurocognitive task where participants are presented with a list of words and perform an immediate recall test. A word recognition task is given following a delay (~−30 min). This task measures encoding, storage, retrieval, and recognition of verbal memory.

Implicit memory: memory that can be recalled without conscious recollection or awareness. (i.e., knowing how to type on a keyboard without looking at the letters).

Multi-voxel pattern analyses: neuroimaging technique that analyzes an individual’s pattern of neural activity during a task to predict their cognitive state or condition.

Neurogenesis: the continuous proliferation of neuronal precursor cells that differentiate into neurons in the mammalian brain (post-natal/adult neurogenesis). Neurogenesis occurs in the sub-granular zone of the dentate gyrus, where neurons integrate into the granule cell layer of the hippocampus. Neurogenesis also occurs in in the subventricular zone, where neurons migrate via the rostral migratory stream to the olfactory bulb.

Paired associate learning: a neurocognitive task in which participants are presented with pairs of words, items, or spatial contexts (i.e., a dog paired with a living room), then later must recognize if the items had previously been presented together, or if the pair is a novel combination (i.e., a dog paired with a beach).

Picture Sequence Memory Test: a standard neurocognitive task from the NIH Toolbox Cognition Battery where participants are presented with a series of pictures and must recall the correct order in which the images were presented. The task is used to measure episodic memory.

Recollection: involves a ‘sense of reliving’, or a re-instantiation of contextual and perceptual details related to the cued event. Recollection is thought to be mediated largely by the hippocampus and parahippocampal cortex.

Recollection network: a network of brain regions which are commonly activated during memory recollection. These regions include the hippocampus, parahippocampus, retrosplenial/posterior parietal cortex, lateral parietal cortex, and the medial prefrontal cortex.

Retrograde memory: memories acquired prior to a particular point in time.

Rey Auditory Verbal Learning Test: a standard neurocognitive task where participants are presented with a list of words and perform an immediate recall test. A word recognition task is given following a delay (~−30 min). This task measures encoding, storage, retrieval, and recognition of verbal memory.

Schema (schematic memory): an associative network of information which is adaptable and developed though the abstraction of common information over the course of multiple episodes (i.e., knowing that a typical birthday party includes balloons, presents, cake, candles). Schemas are thought to be largely represented in the mPFC.

Semantic memory: a component of declarative memory including memory for knowledge about the world and general facts (i.e., Washington D.C. is the capital of the United States of America). Semantic knowledge is largely supported by the anterior temporal lobe.

TEMPau: Test of Episodic Memory of Past Autobiographies; a semi-structured interview that tests one’s memories for unique events occurring at a specific time and place within different lifetime periods.

Trace Transformation Theory (TTT): a theory which proposes that episodic memories are consolidated in rich contextual detail within the hippocampus. The hippocampus is always required for the storage and retrieval of this contextually detailed memory. Over time, episodic memories are transformed into less detailed schematic memories that capture the essential features or gist of the original, and are represented in neocortical regions. The detailed hippocampus-dependent version, and the transformed schematic version of the memory, can co-exist in the brain. The situational demands at the time of retrieval will mediate which version of the memory is expressed.

Voxel-based morphometry: A whole-brain neuroimaging analysis method which determines the regional volume of tissue by measuring the total number of voxels in a region of interest. This method is useful for comparing whole brain tissue volume differences between conditions or groups.

Wechsler Memory Scale-Revised: a standardized neurocognitive test battery that includes logic memory, visual and verbal paired associates, and visual reproduction tasks. These tasks assess numerous cognitive domains including visual, verbal, general, and delayed memory, and attention/concentration.

## References

[1] Wefel J.S., Kesler S.R., Noll K.R., Schagen S.B. (2015). Clinical Characteristics, Pathophysiology, and Management of Noncentral Nervous System Cancer-Related Cognitive Impairment in Adults: Cancer-Related Cognitive Impairment. CA A Cancer J. Clin..

[2] Pullens M.J.J., Vries J.D., Warmerdam L.J.C.V., Wal M.A.V.D., Roukema J.A. (2013). Chemotherapy and Cognitive Complaints in Women with Breast Cancer. Psycho-Oncology.

[3] Ahles T.A., Root J.C., Ryan E.L. (2012). Cancer- and Cancer Treatment–Associated Cognitive Change: An Update on the State of the Science. JCO.

[4] Janelsins M.C., Kesler S.R., Ahles T.A., Morrow G.R. (2014). Prevalence, Mechanisms, and Management of Cancer-Related Cognitive Impairment. Int. Rev. Psychiatry.

[5] Schagen S.B., Muller M.J., Boogerd W., Rosenbrand R.M., van Rhijn D., Rodenhuis S., van Dam F.S.a.M. (2002). Late Effects of Adjuvant Chemotherapy on Cognitive Function: A Follow-up Study in Breast Cancer Patients. Ann. Oncol..

[6] Ahles T.A., Saykin A.J., Furstenberg C.T., Cole B., Mott L.A., Skalla K., Whedon M.B., Bivens S., Mitchell T., Greenberg E.R. (2002). Neuropsychologic Impact of Standard-Dose Systemic Chemotherapy in Long-Term Survivors of Breast Cancer and Lymphoma. JCO.

[7] Hutchinson A.D., Hosking J.R., Kichenadasse G., Mattiske J.K., Wilson C. (2012). Objective and Subjective Cognitive Impairment Following Chemotherapy for Cancer: A Systematic Review. Cancer Treat. Rev..

[8] Janelsins M.C., Kohli S., Mohile S.G., Usuki K., Ahles T.A., Morrow G.R. (2011). An Update on Cancer- and Chemotherapy-Related Cognitive Dysfunction: Current Status. Semin. Oncol..

[9] Ono M., Ogilvie J.M., Wilson J.S., Green H.J., Chambers S.K., Ownsworth T., Shum D.H.K. (2015). A Meta-Analysis of Cognitive Impairment and Decline Associated with Adjuvant Chemotherapy in Women with Breast Cancer. Front. Oncol..

[10] Falleti M.G., Sanfilippo A., Maruff P., Weih L., Phillips K.-A. (2005). The Nature and Severity of Cognitive Impairment Associated with Adjuvant Chemotherapy in Women with Breast Cancer: A Meta-Analysis of the Current Literature. Brain Cogn..

[11] Runowicz C.D., Leach C.R., Henry N.L., Henry K.S., Mackey H.T., Cowens-Alvarado R.L., Cannady R.S., Pratt-Chapman M.L., Edge S.B., Jacobs L.A. (2016). American Cancer Society/American Society of Clinical Oncology Breast Cancer Survivorship Care Guideline. CA Cancer J. Clin..

[12] Von Ah D., Habermann B., Carpenter J.S., Schneider B.L. (2013). Impact of Perceived Cognitive Impairment in Breast Cancer Survivors. Eur. J. Oncol. Nurs..

[13] Koppelmans V., de Ruiter M.B., van der Lijn F., Boogerd W., Seynaeve C., van der Lugt A., Vrooman H., Niessen W.J., Breteler M.M.B., Schagen S.B. (2012). Global and Focal Brain Volume in Long-Term Breast Cancer Survivors Exposed to Adjuvant Chemotherapy. Breast Cancer Res. Treat..

[14] Amidi A., Christensen S., Mehlsen M., Jensen A.B., Pedersen A.D., Zachariae R. (2015). Long-Term Subjective Cognitive Functioning Following Adjuvant Systemic Treatment: 7-9 Years Follow-up of a Nationwide Cohort of Women Treated for Primary Breast Cancer. Br. J. Cancer.

[15] Baxter M.F., Dulworth A.N., Smith T.M. (2011). Identification of Mild Cognitive Impairments in Cancer Survivors. Occup. Ther. Health Care.

[16] Silverman D.H.S., Dy C.J., Castellon S.A., Lai J., Pio B.S., Abraham L., Waddell K., Petersen L., Phelps M.E., Ganz P.A. (2007). Altered Frontocortical, Cerebellar, and Basal Ganglia Activity in Adjuvant-Treated Breast Cancer Survivors 5–10 Years after Chemotherapy. Breast Cancer Res. Treat..

[17] De Ruiter M.B., Reneman L., Boogerd W., Veltman D.J., van Dam F.S.A.M., Nederveen A.J., Boven E., Schagen S.B. (2011). Cerebral Hyporesponsiveness and Cognitive Impairment 10 Years after Chemotherapy for Breast Cancer. Hum. Brain Mapp..

[18] De Ruiter M.B., Reneman L., Boogerd W., Veltman D.J., Caan M., Douaud G., Lavini C., Linn S.C., Boven E., van Dam F.S.A.M. (2012). Late Effects of High-Dose Adjuvant Chemotherapy on White and Gray Matter in Breast Cancer Survivors: Converging Results from Multimodal Magnetic Resonance Imaging. Hum. Brain Mapp..

[19] Stouten-Kemperman M.M., de Ruiter M.B., Koppelmans V., Boogerd W., Reneman L., Schagen S.B. (2015). Neurotoxicity in Breast Cancer Survivors ≥10 Years Post-Treatment Is Dependent on Treatment Type. Brain Imaging Behav..

[20] Castel H., Denouel A., Lange M., Tonon M.-C., Dubois M., Joly F. (2017). Biomarkers Associated with Cognitive Impairment in Treated Cancer Patients: Potential Predisposition and Risk Factors. Front. Pharmacol..

[21] Jean-Pierre P., McDonald B.C. (2016). Neuroepidemiology of Cancer and Treatment-Related Neurocognitive Dysfunction in Adult-Onset Cancer Patients and Survivors. Handb. Clin. Neurol..

[22] Dietrich J., Prust M., Kaiser J. (2015). Chemotherapy, Cognitive Impairment and Hippocampal Toxicity. Neuroscience.

[23] Cheung Y.T., Ng T., Shwe M., Ho H.K., Foo K.M., Cham M.T., Lee J.A., Fan G., Tan Y.P., Yong W.S. (2015). Association of Proinflammatory Cytokines and Chemotherapy-Associated Cognitive Impairment in Breast Cancer Patients: A Multi-Centered, Prospective, Cohort Study. Ann. Oncol..

[24] Limoli C.L., Giedzinski E., Rola R., Otsuka S., Palmer T.D., Fike J.R. (2004). Radiation Response of Neural Precursor Cells: Linking Cellular Sensitivity to Cell Cycle Checkpoints, Apoptosis and Oxidative Stress. Radiat. Res..

[25] Cardoso C.V., de Barros M.P., Bachi A.L.L., Bernardi M.M., Kirsten T.B., de Fátima Monteiro Martins M., Rocha P.R.D., da Silva Rodrigues P., Bondan E.F. (2020). Chemobrain in Rats: Behavioral, Morphological, Oxidative and Inflammatory Effects of Doxorubicin Administration. Behav. Brain Res..

[26] Christie L.-A., Acharya M.M., Parihar V.K., Nguyen A., Martirosian V., Limoli C.L. (2012). Impaired Cognitive Function and Hippocampal Neurogenesis Following Cancer Chemotherapy. Clin. Cancer Res..

[27] Acharya M.M., Martirosian V., Chmielewski N.N., Hanna N., Tran K.K., Liao A.C., Christie L.-A., Parihar V.K., Limoli C.L. (2015). Stem Cell Transplantation Reverses Chemotherapy-Induced Cognitive Dysfunction. Cancer Res..

[28] Groves T.R., Farris R., Anderson J.E., Alexander T.C., Kiffer F., Carter G., Wang J., Boerma M., Allen A.R. (2017). 5-Fluorouracil Chemotherapy Upregulates Cytokines and Alters Hippocampal Dendritic Complexity in Aged Mice. Behav. Brain Res..

[29] Kang S., Lee S., Kim J., Kim J.-C., Kim S.-H., Son Y., Shin T., Youn B., Kim J.-S., Wang H. (2018). Chronic Treatment with Combined Chemotherapeutic Agents Affects Hippocampal Micromorphometry and Function in Mice, Independently of Neuroinflammation. Exp. Neurobiol..

[30] John J., Kinra M., Mudgal J., Viswanatha G.L., Nandakumar K. (2021). Animal Models of Chemotherapy-Induced Cognitive Decline in Preclinical Drug Development. Psychopharmacology.

[31] Mounier N.M., Abdel-Maged A.E.-S., Wahdan S.A., Gad A.M., Azab S.S. (2020). Chemotherapy-Induced Cognitive Impairment (CICI): An Overview of Etiology and Pathogenesis. Life Sci..

[32] Van Praag H., Schinder A.F., Christie B.R., Toni N., Palmer T.D., Gage F.H. (2002). Functional Neurogenesis in the Adult Hippocampus. Nature.

[33] Kee N., Teixeira C.M., Wang A.H., Frankland P.W. (2007). Preferential Incorporation of Adult-Generated Granule Cells into Spatial Memory Networks in the Dentate Gyrus. Nat. Neurosci..

[34] Kitamura T., Saitoh Y., Takashima N., Murayama A., Niibori Y., Ageta H., Sekiguchi M., Sugiyama H., Inokuchi K. (2009). Adult Neurogenesis Modulates the Hippocampus-Dependent Period of Associative Fear Memory. Cell.

[35] Feng R., Rampon C., Tang Y.-P., Shrom D., Jin J., Kyin M., Sopher B., Martin G.M., Kim S.-H., Langdon R.B. (2001). Deficient Neurogenesis in Forebrain-Specific Presenilin-1 Knockout Mice Is Associated with Reduced Clearance of Hippocampal Memory Traces. Neuron.

[36] Martinez-Canabal A., Akers K.G., Josselyn S.A., Frankland P.W. (2013). Age-Dependent Effects of Hippocampal Neurogenesis Suppression on Spatial Learning. Hippocampus.

[37] Epp J.R., Silva Mera R., Köhler S., Josselyn S.A., Frankland P.W. (2016). Neurogenesis-Mediated Forgetting Minimizes Proactive Interference. Nat. Commun..

[38] Shors T.J., Townsend D.A., Zhao M., Kozorovitskiy Y., Gould E. (2002). Neurogenesis May Relate to Some but Not All Types of Hippocampal-Dependent Learning. Hippocampus.

[39] Winocur G., Wojtowicz J.M., Sekeres M., Snyder J.S., Wang S. (2006). Inhibition of Neurogenesis Interferes with Hippocampus-Dependent Memory Function. Hippocampus.

[40] Deng W., Aimone J.B., Gage F.H. (2010). New Neurons and New Memories: How Does Adult Hippocampal Neurogenesis Affect Learning and Memory?. Nat. Rev. Neurosci..

[41] Dupret D., Revest J.-M., Koehl M., Ichas F., Giorgi F.D., Costet P., Abrous D.N., Piazza P.V. (2008). Spatial Relational Memory Requires Hippocampal Adult Neurogenesis. PLoS ONE.

[42] Minotti G., Menna P., Salvatorelli E., Cairo G., Gianni L. (2004). Anthracyclines: Molecular Advances and Pharmacologic Developments in Antitumor Activity and Cardiotoxicity. Pharm. Rev..

[43] El-Agamy S.E., Abdel-Aziz A.K., Esmat A., Azab S.S. (2019). Chemotherapy and Cognition: Comprehensive Review on Doxorubicin-Induced Chemobrain. Cancer Chemother. Pharm..

[44] Monje M.L., Toda H., Palmer T.D. (2003). Inflammatory Blockade Restores Adult Hippocampal Neurogenesis. Science.

[45] Seigers R., Schagen S., Beerling W., Boogerd W., Vantellingen O., Vandam F., Koolhaas J., Buwalda B. (2008). Long-Lasting Suppression of Hippocampal Cell Proliferation and Impaired Cognitive Performance by Methotrexate in the Rat. Behav. Brain Res..

[46] Seigers R., Schagen S.B., Coppens C.M., van der Most P.J., van Dam F.S.A.M., Koolhaas J.M., Buwalda B. (2009). Methotrexate Decreases Hippocampal Cell Proliferation and Induces Memory Deficits in Rats. Behav. Brain Res..

[47] Lyons L., ElBeltagy M., Umka J., Markwick R., Startin C., Bennett G., Wigmore P. (2011). Fluoxetine Reverses the Memory Impairment and Reduction in Proliferation and Survival of Hippocampal Cells Caused by Methotrexate Chemotherapy. Psychopharmacology.

[48] Welbat J.U., Naewla S., Pannangrong W., Sirichoat A., Aranarochana A., Wigmore P. (2020). Neuroprotective Effects of Hesperidin against Methotrexate-Induced Changes in Neurogenesis and Oxidative Stress in the Adult Rat. Biochem. Pharmacol..

[49] Han R., Yang Y.M., Dietrich J., Luebke A., Mayer-Pröschel M., Noble M. (2008). Systemic 5-Fluorouracil Treatment Causes a Syndrome of Delayed Myelin Destruction in the Central Nervous System. J. Biol..

[50] Janelsins M.C., Roscoe J.A., Berg M.J., Thompson B.D., Gallagher M.J., Morrow G.R., Heckler C.E., Jean-Pierre P., Opanashuk L.A., Gross R.A. (2010). IGF-1 Partially Restores Chemotherapy-Induced Reductions in Neural Cell Proliferation in Adult C57BL/6 Mice. Cancer Investig..

[51] Lyons L., ELBeltagy M., Bennett G., Wigmore P. (2012). Fluoxetine Counteracts the Cognitive and Cellular Effects of 5-Fluorouracil in the Rat Hippocampus by a Mechanism of Prevention Rather than Recovery. PLoS ONE.

[52] Sirichoat A., Suwannakot K., Chaisawang P., Pannangrong W., Aranarochana A., Wigmore P., Welbat J.U. (2020). Melatonin Attenuates 5-Fluorouracil-Induced Spatial Memory and Hippocampal Neurogenesis Impairment in Adult Rats. Life Sci..

[53] Dietrich J., Han R., Yang Y., Mayer-Pröschel M., Noble M. (2006). CNS Progenitor Cells and Oligodendrocytes Are Targets of Chemotherapeutic Agents in Vitro and in Vivo. J. Biol..

[54] Manohar S., Jamesdaniel S., Salvi R. (2014). Cisplatin Inhibits Hippocampal Cell Proliferation and Alters the Expression of Apoptotic Genes. Neurotox. Res..

[55] Chiu G.S., Maj M.A., Rizvi S., Dantzer R., Vichaya E.G., Laumet G., Kavelaars A., Heijnen C.J. (2017). Pifithrin-μ Prevents Cisplatin-Induced Chemobrain by Preserving Neuronal Mitochondrial Function. Cancer Res..

[56] Kitamura Y., Hattori S., Yoneda S., Watanabe S., Kanemoto E., Sugimoto M., Kawai T., Machida A., Kanzaki H., Miyazaki I. (2015). Doxorubicin and Cyclophosphamide Treatment Produces Anxiety-like Behavior and Spatial Cognition Impairment in Rats: Possible Involvement of Hippocampal Neurogenesis via Brain-Derived Neurotrophic Factor and Cyclin D1 Regulation. Behav. Brain Res..

[57] Hou J., Xue J., Lee M., Sun M., Zhao X., Zheng Y., Sung C. (2013). Compound K Is Able to Ameliorate the Impaired Cognitive Function and Hippocampal Neurogenesis Following Chemotherapy Treatment. Biochem. Biophys. Res. Commun..

[58] Garthe A., Behr J., Kempermann G. (2009). Adult-Generated Hippocampal Neurons Allow the Flexible Use of Spatially Precise Learning Strategies. PLoS ONE.

[59] Akers K.G., Martinez-Canabal A., Restivo L., Yiu A.P., De Cristofaro A., Hsiang H.-L., Wheeler A.L., Guskjolen A., Niibori Y., Shoji H. (2014). Hippocampal Neurogenesis Regulates Forgetting During Adulthood and Infancy. Science.

[60] Pereira-Caixeta A.R., Guarnieri L.O., Medeiros D.C., Mendes E.M.A.M., Ladeira L.C.D., Pereira M.T., Moraes M.F.D., Pereira G.S. (2018). Inhibiting Constitutive Neurogenesis Compromises Long-Term Social Recognition Memory. Neurobiol. Learn. Mem..

[61] Niibori Y., Yu T.-S., Epp J.R., Akers K.G., Josselyn S.A., Frankland P.W. (2012). Suppression of Adult Neurogenesis Impairs Population Coding of Similar Contexts in Hippocampal CA3 Region. Nat. Commun..

[62] Park H.-S., Kim C.-J., Kwak H.-B., No M.-H., Heo J.-W., Kim T.-W. (2018). Physical Exercise Prevents Cognitive Impairment by Enhancing Hippocampal Neuroplasticity and Mitochondrial Function in Doxorubicin-Induced Chemobrain. Neuropharmacology.

[63] Lee B.E., Choi B.Y., Hong D.K., Kim J.H., Lee S.H., Kho A.R., Kim H., Choi H.C., Suh S.W. (2017). The Cancer Chemotherapeutic Agent Paclitaxel (Taxol) Reduces Hippocampal Neurogenesis via down-Regulation of Vesicular Zinc. Sci. Rep..

[64] Panoz-Brown D., Carey L.M., Smith A.E., Gentry M., Sluka C.M., Corbin H.E., Wu J.-E., Hohmann A.G., Crystal J.D. (2017). The Chemotherapeutic Agent Paclitaxel Selectively Impairs Reversal Learning While Sparing Prior Learning, New Learning and Episodic Memory. Neurobiol. Learn. Mem..

[65] Huehnchen P., Boehmerle W., Springer A., Freyer D., Endres M. (2017). A Novel Preventive Therapy for Paclitaxel-Induced Cognitive Deficits: Preclinical Evidence from C57BL/6 Mice. Transl. Psychiatry.

[66] Briones T.L., Woods J. (2011). Chemotherapy-Induced Cognitive Impairment Is Associated with Decreases in Cell Proliferation and Histone Modifications. BMC Neurosci..

[67] Winocur G., Wojtowicz J.M., Merkley C.M., Tannock I.F. (2016). Environmental Enrichment Protects against Cognitive Impairment Following Chemotherapy in an Animal Model. Behav. Neurosci..

[68] Winocur G., Wojtowicz J.M., Huang J., Tannock I.F. (2014). Physical Exercise Prevents Suppression of Hippocampal Neurogenesis and Reduces Cognitive Impairment in Chemotherapy-Treated Rats. Psychopharmacology.

[69] Winocur G., Wojtowicz J.M., Tannock I.F. (2015). Memory Loss in Chemotherapy-Treated Rats Is Exacerbated in High-Interference Conditions and Related to Suppression of Hippocampal Neurogenesis. Behav. Brain Res..

[70] Winocur G., Berman H., Nguyen M., Binns M.A., Henkelman M., van Eede M., Piquette-Miller M., Sekeres M.J., Wojtowicz J.M., Yu J. (2018). Neurobiological Mechanisms of Chemotherapy-Induced Cognitive Impairment in a Transgenic Model of Breast Cancer. Neuroscience.

[71] Rendeiro C., Sheriff A., Bhattacharya T.K., Gogola J.V., Baxter J.H., Chen H., Helferich W.G., Roy E.J., Rhodes J.S. (2016). Long-Lasting Impairments in Adult Neurogenesis, Spatial Learning and Memory from a Standard Chemotherapy Regimen Used to Treat Breast Cancer. Behav. Brain Res..

[72] Sekeres M.J., Bradley-Garcia M., Martinez-Canabal A., Winocur G. (2021). Chemotherapy-Induced Cognitive Impairment and Hippocampal Neurogenesis: A Review of Physiological Mechanisms and Interventions. IJMS.

[73] Apple A.C., Ryals A.J., Alpert K.I., Wagner L.I., Shih P.-A., Dokucu M., Cella D., Penedo F.J., Voss J.L., Wang L. (2017). Subtle Hippocampal Deformities in Breast Cancer Survivors with Reduced Episodic Memory and Self-Reported Cognitive Concerns. NeuroImage Clin..

[74] Monje M.L., Vogel H., Masek M., Ligon K.L., Fisher P.G., Palmer T.D. (2007). Impaired Human Hippocampal Neurogenesis after Treatment for Central Nervous System Malignancies. Ann. Neurol..

[75] Eriksson P.S., Perfilieva E., Björk-Eriksson T., Alborn A.-M., Nordborg C., Peterson D.A., Gage F.H. (1998). Neurogenesis in the Adult Human Hippocampus. Nat. Med..

[76] Knoth R., Singec I., Ditter M., Pantazis G., Capetian P., Meyer R.P., Horvat V., Volk B., Kempermann G. (2010). Murine Features of Neurogenesis in the Human Hippocampus across the Lifespan from 0 to 100 Years. PLoS ONE.

[77] Spalding K.L., Bergmann O., Alkass K., Bernard S., Salehpour M., Huttner H.B., Boström E., Westerlund I., Vial C., Buchholz B.A. (2013). Dynamics of Hippocampal Neurogenesis in Adult Humans. Cell.

[78] Boldrini M., Fulmore C.A., Tartt A.N., Simeon L.R., Pavlova I., Poposka V., Rosoklija G.B., Stankov A., Arango V., Dwork A.J. (2018). Human Hippocampal Neurogenesis Persists throughout Aging. Cell Stem. Cell.

[79] Kempermann G., Gage F.H., Aigner L., Song H., Curtis M.A., Thuret S., Kuhn H.G., Jessberger S., Frankland P.W., Cameron H.A. (2018). Human Adult Neurogenesis: Evidence and Remaining Questions. Cell Stem. Cell.

[80] Dietrich J., Monje M., Wefel J., Meyers C. (2008). Clinical Patterns and Biological Correlates of Cognitive Dysfunction Associated with Cancer Therapy. Oncologist.

[81] Monje M., Dietrich J. (2012). Cognitive Side Effects of Cancer Therapy Demonstrate a Functional Role for Adult Neurogenesis. Behav. Brain Res..

[82] Nguyen L.D., Ehrlich B.E. (2020). Cellular Mechanisms and Treatments for Chemobrain: Insight from Aging and Neurodegenerative Diseases. EMBO Mol. Med..

[83] Greenaway M.C., Lacritz L.H., Binegar D., Weiner M.F., Lipton A., Munro Cullum C. (2006). Patterns of Verbal Memory Performance in Mild Cognitive Impairment, Alzheimer Disease, and Normal Aging. Cogn. Behav. Neurol..

[84] Lekeu F., Magis D., Marique P., Delbeuck X., Bechet S., Guillaume B., Adam S., Petermans J., Moonen G., Salmon E. (2010). The California Verbal Learning Test and Other Standard Clinical Neuropsychological Tests to Predict Conversion from Mild Memory Impairment to Dementia. J. Clin. Exp. Neuropsychol..

[85] Pliskin J.I., DeDios Stern S., Resch Z.J., Saladino K.F., Ovsiew G.P., Carter D.A., Soble J.R. (2021). Comparing the Psychometric Properties of Eight Embedded Performance Validity Tests in the Rey Auditory Verbal Learning Test, Wechsler Memory Scale Logical Memory, and Brief Visuospatial Memory Test–Revised Recognition Trials for Detecting Invalid Neuropsychological Test Performance. Assessment.

[86] Bonner-Jackson A., Mahmoud S., Miller J., Banks S.J. (2015). Verbal and Non-Verbal Memory and Hippocampal Volumes in a Memory Clinic Population. Alzheimer Res. Ther..

[87] Kelley W.M., Miezin F.M., McDermott K.B., Buckner R.L., Raichle M.E., Cohen N.J., Ollinger J.M., Akbudak E., Conturo T.E., Snyder A.Z. (1998). Hemispheric Specialization in Human Dorsal Frontal Cortex and Medial Temporal Lobe for Verbal and Nonverbal Memory Encoding. Neuron.

[88] Collins B., Mackenzie J., Stewart A., Bielajew C., Verma S. (2009). Cognitive Effects of Hormonal Therapy in Early Stage Breast Cancer Patients: A Prospective Study. Psycho-Oncology.

[89] Collins B., MacKenzie J., Tasca G.A., Scherling C., Smith A. (2014). Persistent Cognitive Changes in Breast Cancer Patients 1 Year Following Completion of Chemotherapy. J. Int. Neuropsychol. Soc..

[90] Ahles T.A., Saykin A.J., McDonald B.C., Li Y., Furstenberg C.T., Hanscom B.S., Mulrooney T.J., Schwartz G.N., Kaufman P.A. (2010). Longitudinal Assessment of Cognitive Changes Associated With Adjuvant Treatment for Breast Cancer: Impact of Age and Cognitive Reserve. JCO.

[91] Menning S., de Ruiter M.B., Veltman D.J., Boogerd W., Oldenburg H.S.A., Reneman L., Schagen S.B. (2017). Changes in Brain Activation in Breast Cancer Patients Depend on Cognitive Domain and Treatment Type. PLoS ONE.

[92] Gilboa A., Moscovitch M. (2021). No Consolidation without Representation: Correspondence between Neural and Psychological Representations in Recent and Remote Memory. Neuron.

[93] Tulving E. (1972). Episodic and Semantic Memory. Organization of Memory.

[94] Patterson K., Nestor P.J., Rogers T.T. (2007). Where Do You Know What You Know? The Representation of Semantic Knowledge in the Human Brain. Nat. Rev. Neurosci..

[95] Sekeres M.J., Winocur G., Moscovitch M. (2018). The Hippocampus and Related Neocortical Structures in Memory Transformation. Neurosci. Lett..

[96] Tulving E. (1985). Memory and Consciousness. Can. Psychol. Psychol. Can..

[97] Rosenbaum R.S., Köhler S., Schacter D.L., Moscovitch M., Westmacott R., Black S.E., Gao F., Tulving E. (2005). The Case of K.C.: Contributions of a Memory-Impaired Person to Memory Theory. Neuropsychologia.

[98] Tulving E. (2002). Episodic Memory: From Mind to Brain. Annu. Rev. Psychol..

[99] Steinvorth S., Levine B., Corkin S. (2005). Medial Temporal Lobe Structures Are Needed to Re-Experience Remote Autobiographical Memories: Evidence from H.M. and W.R. Neuropsychologia.

[100] St-Laurent M., Moscovitch M., Levine B., McAndrews M.P. (2009). Determinants of Autobiographical Memory in Patients with Unilateral Temporal Lobe Epilepsy or Excisions. Neuropsychologia.

[101] Conway M.A., Cohen G., Stanhope N. (1991). On the Very Long-Term Retention of Knowledge Acquired through Formal Education: Twelve Years of Cognitive Psychology. J. Exp. Psychol. Gen..

[102] Berntsen D. (2002). Tunnel Memories for Autobiographical Events: Central Details Are Remembered More Frequently from Shocking than from Happy Experiences. Mem. Cogn..

[103] Inagaki M., Matsuoka Y., Sugahara Y., Nakano T., Akechi T., Fujimori M., Imoto S., Murakami K., Uchitomi Y. (2004). Hippocampal Volume and First Major Depressive Episode After Cancer Diagnosis in Breast Cancer Survivors. AJP.

[104] Yoshikawa E., Matsuoka Y., Inagaki M., Nakano T., Akechi T., Kobayakawa M., Fujimori M., Nakaya N., Akizuki N., Imoto S. (2005). No Adverse Effects of Adjuvant Chemotherapy on Hippocampal Volumein Japanese Breast Cancer Survivors. Breast Cancer Res. Treat.

[105] Ferguson R.J., McDonald B.C., Saykin A.J., Ahles T.A. (2007). Brain Structure and Function Differences in Monozygotic Twins: Possible Effects of Breast Cancer Chemotherapy. JCO.

[106] Inagaki M., Yoshikawa E., Matsuoka Y., Sugawara Y., Nakano T., Akechi T., Wada N., Imoto S., Murakami K., Uchitomi Y. (2007). Smaller Regional Volumes of Brain Gray and White Matter Demonstrated in Breast Cancer Survivors Exposed to Adjuvant Chemotherapy. Cancer.

[107] McDonald B.C., Conroy S.K., Ahles T.A., West J.D., Saykin A.J. (2010). Gray Matter Reduction Associated with Systemic Chemotherapy for Breast Cancer: A Prospective MRI Study. Breast Cancer Res. Treat.

[108] Conroy S.K., McDonald B.C., Smith D.J., Moser L.R., West J.D., Kamendulis L.M., Klaunig J.E., Champion V.L., Unverzagt F.W., Saykin A.J. (2013). Alterations in Brain Structure and Function in Breast Cancer Survivors: Effect of Post-Chemotherapy Interval and Relation to Oxidative DNA Damage. Breast Cancer Res. Treat.

[109] Kesler S., Janelsins M., Koovakkattu D., Palesh O., Mustian K., Morrow G., Dhabhar F.S. (2013). Reduced Hippocampal Volume and Verbal Memory Performance Associated with Interleukin-6 and Tumor Necrosis Factor-Alpha Levels in Chemotherapy-Treated Breast Cancer Survivors. Brain Behav. Immun..

[110] Lepage C., Smith A.M., Moreau J., Barlow-Krelina E., Wallis N., Collins B., MacKenzie J., Scherling C. (2014). A Prospective Study of Grey Matter and Cognitive Function Alterations in Chemotherapy-Treated Breast Cancer Patients. SpringerPlus.

[111] Kesler S.R., Bennett F.C., Mahaffey M.L., Spiegel D. (2009). Regional Brain Activation during Verbal Declarative Memory in Metastatic Breast Cancer. Clin. Cancer Res..

[112] López Zunini R.A., Scherling C., Wallis N., Collins B., MacKenzie J., Bielajew C., Smith A.M. (2013). Differences in Verbal Memory Retrieval in Breast Cancer Chemotherapy Patients Compared to Healthy Controls: A Prospective FMRI Study. Brain Imaging Behav..

[113] Apple A.C., Schroeder M.P., Ryals A.J., Wagner L.I., Cella D., Shih P.-A., Reilly J., Penedo F.J., Voss J.L., Wang L. (2018). Hippocampal Functional Connectivity Is Related to Self-Reported Cognitive Concerns in Breast Cancer Patients Undergoing Adjuvant Therapy. NeuroImage Clin..

[114] Bruno J., Hosseini S.M.H., Kesler S. (2012). Altered Resting State Functional Brain Network Topology in Chemotherapy-Treated Breast Cancer Survivors. Neurobiol. Dis..

[115] Tao L., Lin H., Yan Y., Xu X., Wang L., Zhang J., Yu Y. (2017). Impairment of the Executive Function in Breast Cancer Patients Receiving Chemotherapy Treatment: A Functional MRI Study. Eur. J. Cancer Care.

[116] Cheng H., Li W., Gong L., Xuan H., Huang Z., Zhao H., Wang L.S., Wang K. (2017). Altered Resting-State Hippocampal Functional Networks Associated with Chemotherapy-Induced Prospective Memory Impairment in Breast Cancer Survivors. Sci. Rep..

[117] Chen Y., Ou Y., Lv D., Yang R., Li S., Jia C., Wang Y., Meng X., Cui H., Li C. (2019). Altered Network Homogeneity of the Default-Mode Network in Drug-Naive Obsessive−compulsive Disorder. Prog. Neuro-Psychopharmacol. Biol. Psychiatry.

[118] Feng Y., Tuluhong D., Shi Z., Zheng L.J., Chen T., Lu G.M., Wang S., Zhang L.J. (2020). Postchemotherapy Hippocampal Functional Connectivity Patterns in Patients with Breast Cancer: A Longitudinal Resting State Functional MR Imaging Study. Brain Imaging Behav..

[119] Feng Y., Wang Y.F., Zheng L.J., Shi Z., Huang W., Zhang L.J. (2020). Network-Level Functional Connectivity Alterations in Chemotherapy Treated Breast Cancer Patients: A Longitudinal Resting State Functional MRI Study. Cancer Imaging.

[120] Bonnici H.M., Chadwick M.J., Lutti A., Hassabis D., Weiskopf N., Maguire E.A. (2012). Detecting Representations of Recent and Remote Autobiographical Memories in VmPFC and Hippocampus. J. Neurosci..

[121] Bonnici H.M., Maguire E.A. (2018). Two Years Later–Revisiting Autobiographical Memory Representations in VmPFC and Hippocampus. Neuropsychologia.

[122] Boccia M., Teghil A., Guariglia C. (2019). Looking into Recent and Remote Past: Meta-Analytic Evidence for Cortical Re-Organization of Episodic Autobiographical Memories. Neurosci. Biobehav. Rev..

[123] Saxe M.D., Battaglia F., Wang J.-W., Malleret G., David D.J., Monckton J.E., Garcia A.D.R., Sofroniew M.V., Kandel E.R., Santarelli L. (2006). Ablation of Hippocampal Neurogenesis Impairs Contextual Fear Conditioning and Synaptic Plasticity in the Dentate Gyrus. Proc. Natl. Acad. Sci. USA.

[124] Drew M.R., Denny C.A., Hen R. (2010). Arrest of Adult Hippocampal Neurogenesis in Mice Impairs Single- but Not Multiple-Trial Contextual Fear Conditioning. Behav. Neurosci..

[125] Gurguryan L., Rioux M., Sheldon S. (2021). Reduced Anterior Hippocampal and Ventromedial Prefrontal Activity When Repeatedly Retrieving Autobiographical Memories. Hippocampus.

[126] Mccormick C., St-Laurent M., Ty A., Valiante T., Mcandrews M. (2013). Functional and Effective Hippocampal-Neocortical Connectivity During Construction and Elaboration of Autobiographical Memory Retrieval. Cereb. Cortex.

[127] St-Laurent M., Moscovitch M., Jadd R., McAndrews M.P. (2014). The Perceptual Richness of Complex Memory Episodes Is Compromised by Medial Temporal Lobe Damage. Hippocampus.

[128] Rosenbaum R.S., Moscovitch M., Foster J.K., Schnyer D.M., Gao F., Kovacevic N., Verfaellie M., Black S.E., Levine B. (2008). Patterns of Autobiographical Memory Loss in Medial-Temporal Lobe Amnesic Patients. J. Cogn. Neurosci..

[129] Nilsson-Ihrfelt E., Fjällskog M.-L., Liss A., Jakobsson O., Blomqvist C., Andersson G. (2004). Autobiographical Memories in Patients Treated for Breast Cancer. J. Psychosom. Res..

[130] Conway M.A., Pleydell-Pearce C.W. (2000). The Construction of Autobiographical Memories in the Self-Memory System. Psychol. Rev..

[131] Williams J.M., Broadbent K. (1986). Autobiographical Memory in Suicide Attempters. J. Abnorm. Psychol..

[132] Bergouignan L., Lefranc J.P., Chupin M., Morel N., Spano J.P., Fossati P. (2011). Breast Cancer Affects Both the Hippocampus Volume and the Episodic Autobiographical Memory Retrieval. PLoS ONE.

[133] Sekeres M.J., Winocur G., Moscovitch M., Anderson J.A.E., Pishdadian S., Martin Wojtowicz J., St-Laurent M., McAndrews M.P., Grady C.L. (2018). Changes in Patterns of Neural Activity Underlie a Time-Dependent Transformation of Memory in Rats and Humans. Hippocampus.

[134] Sekeres M.J., Moscovitch M., Winocur G., Pishdadian S., Nichol D., Grady C.L. (2021). Reminders Activate the Prefrontal-medial Temporal Cortex and Attenuate Forgetting of Event Memory. Hippocampus.

[135] Kopelman M.D., Wilson B.A., Baddeley A.D. (1989). The Autobiographical Memory Interview: A New Assessment of Autobiographical and Personal Semantic Memory in Amnesic Patients. J. Clin. Exp. Neuropsychol..

[136] Levine B., Svoboda E., Hay J.F., Winocur G., Moscovitch M. (2002). Aging and Autobiographical Memory: Dissociating Episodic from Semantic Retrieval. Psychol. Aging.

[137] Addis D.R., Wong A.T., Schacter D.L. (2007). Remembering the Past and Imagining the Future: Common and Distinct Neural Substrates during Event Construction and Elaboration. Neuropsychologia.

[138] Dede A.J.O., Frascino J.C., Wixted J.T., Squire L.R. (2016). Learning and Remembering Real-World Events after Medial Temporal Lobe Damage. Proc. Natl. Acad. Sci. USA.

[139] Miller T.D., Chong T.T.-J., Aimola Davies A.M., Johnson M.R., Irani S.R., Husain M., Ng T.W., Jacob S., Maddison P., Kennard C. (2020). Human Hippocampal CA3 Damage Disrupts Both Recent and Remote Episodic Memories. eLife.

[140] Peters S., Sheldon S. (2020). Interindividual Differences in Cognitive Functioning Are Associated with Autobiographical Memory Retrieval Specificity in Older Adults. GeroPsych.

[141] Wank A.A., Andrews-Hanna J.R., Grilli M.D. (2021). Searching for the Past: Exploring the Dynamics of Direct and Generative Autobiographical Memory Reconstruction among Young and Cognitively Normal Older Adults. Mem. Cogn..

[142] Sekeres M.J., Riggs L., Decker A., de Medeiros C.B., Bacopulos A., Skocic J., Szulc-Lerch K., Bouffet E., Levine B., Grady C.L. (2018). Impaired Recent, but Preserved Remote, Autobiographical Memory in Pediatric Brain Tumor Patients. J. Neurosci..

[143] Sekeres M.J., Bonasia K., St-Laurent M., Pishdadian S., Winocur G., Grady C., Moscovitch M. (2016). Recovering and Preventing Loss of Detailed Memory: Differential Rates of Forgetting for Detail Types in Episodic Memory. Learn. Mem..

[144] Nakano T., Wenner M., Inagaki M., Kugaya A., Akechi T., Matsuoka Y., Sugahara Y., Imoto S., Murakami K., Uchitomi Y. (2002). Relationship Between Distressing Cancer-Related Recollections and Hippocampal Volume in Cancer Survivors. AJP.

[145] Deprez S., Amant F., Smeets A., Peeters R., Leemans A., Van Hecke W., Verhoeven J.S., Christiaens M.-R., Vandenberghe J., Vandenbulcke M. (2012). Longitudinal Assessment of Chemotherapy-Induced Structural Changes in Cerebral White Matter and Its Correlation With Impaired Cognitive Functioning. JCO.

[146] McDonald B.C., Conroy S.K., Ahles T.A., West J.D., Saykin A.J. (2012). Alterations in Brain Activation During Working Memory Processing Associated With Breast Cancer and Treatment: A Prospective Functional Magnetic Resonance Imaging Study. J. Clin. Oncol..

[147] Poppenk J., Evensmoen H.R., Moscovitch M., Nadel L. (2013). Long-Axis Specialization of the Human Hippocampus. Trends Cogn. Sci..

[148] Fanselow M.S., Dong H.-W. (2010). Are the Dorsal and Ventral Hippocampus Functionally Distinct Structures?. Neuron.

[149] Moscovitch M., Cabeza R., Winocur G., Nadel L. (2016). Episodic Memory and Beyond: The Hippocampus and Neocortex in Transformation. Annu. Rev. Psychol..

[150] Poppenk J., Moscovitch M. (2011). A Hippocampal Marker of Recollection Memory Ability among Healthy Young Adults: Contributions of Posterior and Anterior Segments. Neuron.

[151] Robin J., Moscovitch M. (2017). Details, Gist and Schema: Hippocampal–Neocortical Interactions Underlying Recent and Remote Episodic and Spatial Memory. Curr. Opin. Behav. Sci..

[152] Inman C.S., James G.A., Vytal K., Hamann S. (2018). Dynamic Changes in Large-Scale Functional Network Organization during Autobiographical Memory Retrieval. Neuropsychologia.

[153] Bauer P.J., Dikmen S.S., Heaton R.K., Mungas D., Slotkin J., Beaumont J.L. (2013). III. NIH Toolbox Cognition Battery (CB): Measuring Episodic Memory: NIH Toolbox Cognition Battery (CB). Monogr. Soc. Res. Child.

[154] Ryals A.J., Apple A.C., Wang J.X., Cella D., Penedo F.J., Wang L., Voss J.L. (2015). Hippocampal Memory Impairment in Breast Cancer Survivors after Chemotherapy Measurement Using Covert Testing. JCO.

[155] Schacter D.L. (1987). Implicit Memory: History and Current Status. J. Exp. Psychol. Learn. Mem. Cogn..

[156] Rugg M.D., Mark R.E., Walla P., Schloerscheidt A.M., Birch C.S., Allan K. (1998). Dissociation of the Neural Correlates of Implicit and Explicit Memory. Nature.

[157] Buckner R.L., Goodman J., Burock M., Rotte M., Koutstaal W., Schacter D., Rosen B., Dale A.M. (1998). Functional-Anatomic Correlates of Object Priming in Humans Revealed by Rapid Presentation Event-Related FMRI. Neuron.

[158] Raichle M.E., MacLeod A.M., Snyder A.Z., Powers W.J., Gusnard D.A., Shulman G.L. (2001). A Default Mode of Brain Function. Proc. Natl. Acad. Sci. USA.

[159] Buckner R.L., Andrews-Hanna J.R., Schacter D.L. (2008). The Brain’s Default Network: Anatomy, Function, and Relevance to Disease. Ann. N. Y. Acad. Sci..

[160] Spreng R.N., Mar R.A., Kim A.S.N. (2009). The Common Neural Basis of Autobiographical Memory, Prospection, Navigation, Theory of Mind, and the Default Mode: A Quantitative Meta-Analysis. J. Cogn. Neurosci..

[161] Buckner R.L., Carroll D.C. (2007). Self-Projection and the Brain. Trends Cogn. Sci..

[162] Andrews-Hanna J.R., Reidler J.S., Sepulcre J., Poulin R., Buckner R.L. (2010). Functional-Anatomic Fractionation of the Brain’s Default Network. Neuron.

[163] Spreng R.N., Grady C.L. (2010). Patterns of Brain Activity Supporting Autobiographical Memory, Prospection, and Theory of Mind, and Their Relationship to the Default Mode Network. J. Cogn. Neurosci..

[164] Okuda J., Fujii T., Ohtake H., Tsukiura T., Tanji K., Suzuki K., Kawashima R., Fukuda H., Itoh M., Yamadori A. (2003). Thinking of the Future and Past: The Roles of the Frontal Pole and the Medial Temporal Lobes. Neuroimage.

[165] Addis D.R., Schacter D.L. (2011). The Hippocampus and Imagining the Future: Where Do We Stand?. Front. Hum. Neurosci..

[166] Schacter D.L., Addis D.R., Buckner R.L. (2007). Remembering the Past to Imagine the Future: The Prospective Brain. Nat. Rev. Neurosci..

[167] Hassabis D., Maguire E.A. (2009). The Construction System of the Brain. Philos. Trans. R. Soc. B Biol. Sci..

[168] Schacter D.L., Addis D.R. (2007). The Ghosts of Past and Future. Nature.

[169] Schacter D.L. (2012). Adaptive Constructive Processes and the Future of Memory. Am. Psychol..

[170] McDaniel M.A., Glisky E.L., Guynn M.J., Routhieaux B.C. (1999). Prospective Memory: A Neuropsychological Study. Neuropsychology.

[171] Burgess P.W., Quayle A., Frith C.D. (2001). Brain Regions Involved in Prospective Memory as Determined by Positron Emission Tomography. Neuropsychologia.

[172] Cona G., Scarpazza C., Sartori G., Moscovitch M., Bisiacchi P.S. (2015). Neural Bases of Prospective Memory: A Meta-Analysis and the “Attention to Delayed Intention” (AtoDI) Model. Neurosci. Biobehav. Rev..

[173] McDaniel M.A., Einstein G.O. (2011). The Neuropsychology of Prospective Memory in Normal Aging: A Componential Approach. Neuropsychologia.

[174] Cheng H., Yang Z., Dong B., Chen C., Zhang M., Huang Z., Chen Z., Wang K. (2013). Chemotherapy-Induced Prospective Memory Impairment in Patients with Breast Cancer: Chemotherapy-Induced PM Impairment in Breast Cancer Patients. Psycho-Oncology.

[175] Bedard M., Verma S., Collins B., Song X., Paquet L. (2016). Prospective Memory Impairment in Chemotherapy-Exposed Early Breast Cancer Survivors: Preliminary Evidence from a Clinical Test. J. Psychosoc. Oncol..

[176] Atance C.M., O’Neill D.K. (2001). Episodic Future Thinking. Trends Cogn. Sci..

[177] Tulving E., Roediger H.L., Nairne J.S., Neath I., Surprenant A.M. (2001). Origin of Autonoesis in Episodic Memory. The Nature of Remembering: Essays in Honor of Robert G. Crowder.

[178] Schacter D.L., Benoit R.G., Szpunar K.K. (2017). Episodic Future Thinking: Mechanisms and Functions. Curr. Opin. Behav. Sci..

[179] Gilboa A., Winocur G., Rosenbaum R.S., Poreh A., Gao F., Black S.E., Westmacott R., Moscovitch M. (2006). Hippocampal Contributions to Recollection in Retrograde and Anterograde Amnesia. Hippocampus.

[180] Race E., Keane M.M., Verfaellie M. (2011). Medial Temporal Lobe Damage Causes Deficits in Episodic Memory and Episodic Future Thinking Not Attributable to Deficits in Narrative Construction. J. Neurosci..

[181] De Luca F., Benuzzi F., Bertossi E., Braghittoni D., di Pellegrino G., Ciaramelli E. (2018). Episodic Future Thinking and Future-Based Decision-Making in a Case of Retrograde Amnesia. Neuropsychologia.

[182] McCormick C., Rosenthal C.R., Miller T.D., Maguire E.A. (2018). Mind-Wandering in People with Hippocampal Damage. J. Neurosci..

[183] Kesler S.R., Adams M., Packer M., Rao V., Henneghan A.M., Blayney D.W., Palesh O. (2017). Disrupted Brain Network Functional Dynamics and Hyper-Correlation of Structural and Functional Connectome Topology in Patients with Breast Cancer Prior to Treatment. Brain Behav..

[184] Kesler S.R., Wefel J.S., Hosseini S.M.H., Cheung M., Watson C.L., Hoeft F. (2013). Default Mode Network Connectivity Distinguishes Chemotherapy-Treated Breast Cancer Survivors from Controls. Proc. Natl. Acad. Sci. USA.

[185] Norman K.A., Polyn S.M., Detre G.J., Haxby J.V. (2006). Beyond Mind-Reading: Multi-Voxel Pattern Analysis of FMRI Data. Trends Cogn. Sci..

[186] Kesler S.R. (2014). Default Mode Network as a Potential Biomarker of Chemotherapy-Related Brain Injury. Neurobiol. Aging.

[187] Wang L., Yan Y., Wang X., Tao L., Chen Q., Bian Y., He X., Liu Y., Ding W., Yu Y. (2016). Executive Function Alternations of Breast Cancer Patients After Chemotherapy. Acad. Radiol..

[188] Miao H., Chen X., Yan Y., He X., Hu S., Kong J., Wu M., Wei Y., Zhou Y., Wang L. (2016). Functional Connectivity Change of Brain Default Mode Network in Breast Cancer Patients after Chemotherapy. Neuroradiology.

[189] Smith A.M., Leeming A., Fang Z., Hatchard T., Mioduszewski O., Schneider M.A., Ferdossifard A., Shergill Y., Khoo E.-L., Poulin P. (2021). Mindfulness-Based Stress Reduction Alters Brain Activity for Breast Cancer Survivors with Chronic Neuropathic Pain: Preliminary Evidence from Resting-State FMRI. J. Cancer Surviv..

[190] Menning S., de Ruiter M.B., Veltman D.J., Koppelmans V., Kirschbaum C., Boogerd W., Reneman L., Schagen S.B. (2015). Multimodal MRI and Cognitive Function in Patients with Breast Cancer Prior to Adjuvant Treatment—The Role of Fatigue. NeuroImage Clin..

[191] Scherling C., Collins B., MacKenzie J., Bielajew C., Smith A. (2012). Prechemotherapy Differences in Response Inhibition in Breast Cancer Patients Compared to Controls: A Functional Magnetic Resonance Imaging Study. J. Clin. Exp. Neuropsychol..

[192] Cimprich B., Reuter-Lorenz P., Nelson J., Clark P.M., Therrien B., Normolle D., Berman M.G., Hayes D.F., Noll D.C., Peltier S. (2010). Prechemotherapy Alterations in Brain Function in Women with Breast Cancer. J. Clin. Exp. Neuropsychol..

[193] Vardy J.L., Stouten-Kemperman M.M., Pond G., Booth C.M., Rourke S.B., Dhillon H.M., Dodd A., Crawley A., Tannock I.F. (2019). A Mechanistic Cohort Study Evaluating Cognitive Impairment in Women Treated for Breast Cancer. Brain Imaging Behav..

[194] Qin S., van Marle H.J.F., Hermans E.J., Fernández G. (2011). Subjective Sense of Memory Strength and the Objective Amount of Information Accurately Remembered Are Related to Distinct Neural Correlates at Encoding. J. Neurosci..

[195] Johnson M.K., Kuhl B.A., Mitchell K.J., Ankudowich E., Durbin K.A. (2015). Age-Related Differences in the Neural Basis of the Subjective Vividness of Memories: Evidence from Multivoxel Pattern Classification. Cogn. Affect. Behav. Neurosci..

[196] Loftus E. (1979). Eyewitness Reliability. Science.

[197] Bender C.M., Sereika S.M., Berga S.L., Vogel V.G., Brufsky A.M., Paraska K.K., Ryan C.M. (2006). Cognitive Impairment Associated with Adjuvant Therapy in Breast Cancer. Psycho-Oncology.

[198] Balash Y., Mordechovich M., Shabtai H., Giladi N., Gurevich T., Korczyn A.D. (2013). Subjective Memory Complaints in Elders: Depression, Anxiety, or Cognitive Decline?. Acta Neurol. Scand..

[199] Abbey G., Thompson S.B.N., Hickish T., Heathcote D. (2015). A Meta-analysis of Prevalence Rates and Moderating Factors for Cancer-related Post-traumatic Stress Disorder. Psycho-Oncology.

[200] Dimitrov L., Moschopoulou E., Korszun A. (2019). Interventions for the Treatment of Cancer-related Traumatic Stress Symptoms: A Systematic Review of the Literature. Psycho-Oncology.

[201] Sebri V., Triberti S., Pravettoni G. (2020). Injured Self: Autobiographical Memory, Self-Concept, and Mental Health Risk in Breast Cancer Survivors. Front. Psychol..

[202] Conway M.A. (2005). Memory and the Self☆. J. Mem. Lang..

[203] Morel N., Dayan J., Piolino P., Viard A., Allouache D., Noal S., Levy C., Joly F., Eustache F., Giffard B. (2015). Emotional Specificities of Autobiographical Memory after Breast Cancer Diagnosis. Conscious. Cogn..

